# Silver Oxide Reduction Chemistry in an Alcohol Environment

**DOI:** 10.1021/acsomega.6c03865

**Published:** 2026-06-23

**Authors:** Fayez A. Alfayez, Simon Ducolombier, Walter R. Caseri, Qun Ren, Sabyasachi Gaan, Manfred Heuberger

**Affiliations:** † Advanced Fibers, Empa Swiss Federal Laboratories for Materials Science and Technology, St Gallen CH-9014, Switzerland; ‡ Department of Materials, ETH Zürich, Zürich CH-8093, Switzerland; § Laboratory for Biointerfaces, 111825 Empa Swiss Federal Laboratories for Materials Science and Technology, St Gallen CH-9014, Switzerland

## Abstract

The polymer-assisted in situ thermal reduction of metal oxides is a promising, one-step method for generating polymer nanoparticle composites; however, the fundamental mechanisms governing nanoparticle formation within polymer melts remain poorly understood. In this work, systematic studies were carried out to elucidate how silver oxide (Ag_2_O) is reduced during compounding with poly­(vinyl alcohol) (PVA). While initial extrusion trials successfully demonstrated the reduction of Ag_2_O, characterizing the precise reaction pathways directly within the complex extrusion system proved difficult. To overcome this, various model liquid systems were employed, namely 1-decanol, 4-decanol, and 2,4-pentanediol. For this model system methodology, standard analytical instrumentation was employed, including DSC, GC-TCD, GC-MS, FTIR spectroscopy, SEM-EDX spectroscopy, and KFT, to comprehensively evaluate the reaction mechanisms. Quantitative analysis of the H_2_O and CO_2_ byproducts revealed that the redox reaction is highly temperature-dependent and fundamentally limited by system mobility. Furthermore, the position of the hydroxyl group in the reducing agent significantly influenced the reaction pathway, dictating the balance between low-temperature oxidative dehydrogenation and high-temperature complete oxidation. It was also found that the morphology of the reduced Ag is dominated by a surface-solid transformation reaction, with Ag mobility slightly influenced by in situ H_2_O generation. Consequently, we propose that enhancing system mobility, either through a low melting temperature PVA or through a more soluble precursor, could overcome inherent solid–solid diffusion limitations, thereby improving nanoparticle dispersion and minimizing agglomeration. Finally, to demonstrate the material’s application potential once mobility limitations are overcome, idealized well-dispersed PVA/Ag nanocomposite films were fabricated via solution casting, exhibiting distinct dichroism and robust antimicrobial properties.

## Graphical abstract

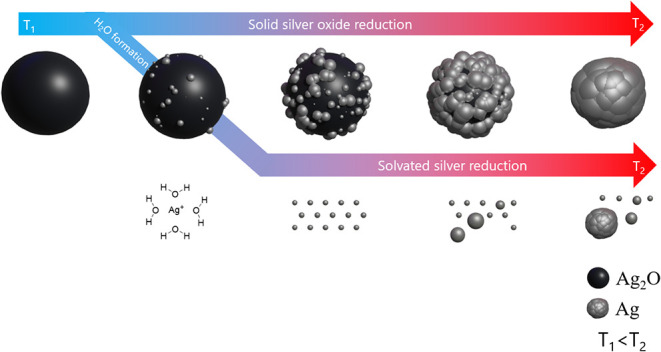

## 1 Introduction

Nanostructured materials have found their way into many application fields over the last few decades, driven by the availability of novel physical and chemical properties and related characterization methods.^1^ The functionality of an important class of hybrid materials relies on a distinct division between an inorganic or metallic phase inside an organic matrix. Synthetic polymers are useful organic matrices, offering advantages such as lightweight, corrosion resistance, ease of processing, and scalable production. The incorporation of metallic phases into such polymeric matrices can yield composite systems with hybrid properties, for example, materials that can conduct heat and electricity, display optical activity, have high dielectric constants or exhibit antimicrobial properties.^2^

The intimate combination of metals and polymers is achieved through diverse processing strategies. While surface deposition techniques such as plating, vacuum deposition, and cold spraying^3,4^ can coat the exterior of formed components, a bulk integration is often accomplished via physical melt compounding.^5^ In such processes, previously synthesized and preconditioned metal is often blended into an existing molten matrix under shear. Alternatively, in situ polymerization allows for a simultaneous generation of the metallic phase during the polymer chain formation.^6^ Solution-based approaches such as solvent casting and sol–gel synthesis are also utilized to achieve nanoscale mixing; in a related approach, solvent impregnation facilitates the infiltration of metal ions into solid preformed polymer networks.

The polymer melt-assisted thermal reduction of metal precursors stands out as an elegant technique for its low chemical waste production and scalability.^5,7^ This in situ method relies on the chemically reducing effect that some organic matrices have on metal oxide at elevated temperatures. Compounding metal salts directly with polymers such as polyamide 6 (PA6), polypropylene (PP), or poly­(lactic acid) (PLA) during the melt-processing step yields very uniform distribution of metal particles throughout the bulk. Many suitable metal oxide salts are available and the reduction reaction yields noncorrosive byproducts; this is different when using common metal nitrates or acetates, which can provoke corrosion and damage to processing equipment.^8^

Despite its established status and the technical simplifications accomplished by such reactive extrusion, a deeper understanding of the underlying chemistry remains remarkably unclear. This can be attributed to the difficulty of monitoring reaction kinetics in situ; the polymer melt is a complex physical environment characterized by high viscosity, shear forces, diffusion, and thermal gradients.^9^ These physical factors complicate fundamental insights into chemical mechanisms, making it difficult to isolate how reduction initiation, metal particle growth, and morphological shaping occur. Several studies have indeed explored the use of reactive extrusion,^10,11^ yet there is still a need for a more detailed understanding to better control the material’s final properties.

Further, the prospect of better controlling the metal particle morphology is of high application relevance. From an application point of view, anisotropic nanoparticles embedded in polymer films are utilized for antimicrobial applications, while aligned nanoparticles can exhibit anisotropic optical properties within the polymer matrix.^24,25^

To decouple the complex physics of the melt extrusion process from the reduction chemistry, we employ a model liquid approach, where we utilize small molecule liquids (i.e., low molecular-weight melts) of selected chemistry to mimic the specific functional groups of the polymer backbone.^12^ Poly­(vinyl alcohol) (PVA) was selected as the host polymer matrix here to study the chemistry related to its hydroxyl-dense backbone, which is known to act as a reducing agent.^13^ In solution-based analogous model systems, we validated monoalcohols and a diol as suitable low-melting models.

Indeed, in solution-based systems, diols^13^ or monoalcohols^14^ are already established as reducing agents and major chemical pathways were investigated. While specific pathways may vary, the mechanism is fundamentally ionic, involving stepwise oxidation of the alcohol to CO_2._ ^14^ This process releases electrons and water byproducts in every step, under concomitant reduction of the metal ion. Regarding the electron transfer step, the reduction is often attributed to the release of a hydride ion (H^–^);^15–17^ however, recent studies demonstrate that under alkaline conditions, alkoxide ions (RO^–^) function as direct electron donors to metal cations.^18^

While PLA/Ag,^7,19^ PP/Ag,^20^ PE/Ag,^20^ PA6/Ag,^21^ EVOH/Ag^22^ nanocomposites have been synthesized via melt extrusion, and others incorporate PEG,^11^ PVP,^11^ or PVA^23^ as reducing agents, there are yet limited extrusion mechanism studies. In contrast to the aforementioned existing literature that relies on solid-state extrusion systems, which frequently suffer from unwanted side reactions, thermal degradation, and complex oxygen diffusion, a notable particularity of this study lies in the use of model liquids.

Furthermore, Ag was chosen as the metallic component for its versatile functionality, while silver­(I) oxide (Ag_2_O) served as the optimal precursor due to its high thermal stability, high metal loading, and nontoxic byproducts. To the best of our knowledge, the reaction mechanism of Ag_2_O with alcohol has been scarcely studied. Ag_2_O is reduced to elemental Ag at 400 °C^12^; however, the addition of glycol to Ag_2_O significantly lowers this reduction temperature.^26^ Thus, Ag_2_O-glycol mixtures have been used as a lead-free soldering substance for electronic packaging to join metals^26–28^ and silicon-based materials.^29–31^ Moreover, Ag_2_O has been used as a catalyst in the decomposition of ozone^32^ and as a primary component in Ag_2_O-zinc batteries. In organic synthesis, Ag_2_O serves as a mild oxidizing agent capable of converting aldehydes into carboxylic acids at room temperature.

In this work, we first compound PVA with Ag_2_O to frame a general understanding of the relevant mechanisms, then we perform a comprehensive kinetic and thermal study on selected model liquids to individually identify the reaction pathways and byproducts. Namely, three specific model liquids were selected: 1-decanol to represent primary alcohols, 4-decanol to represent isolated secondary alcohols, and 2,4-pentanediol to represent the specific steric and electronic structure of the PVA chain. By correlating these findings with observations obtained in the PVA melt compounding process, we aim to provide a mechanistic description of Ag formation during compounding. We propose that for the redox reaction to propagate through the bulk, a mobile phase of either metal or alcohol functionality is essential, requiring either the polymer to be in a molten state or the metal to be as a solvated species. Consequently, solvent-free polymer melts with analysis of mobile metal species are comparatively investigated. This study concludes by demonstrating the fabrication of nanoparticulate polymer films with targeted optical and antibacterial properties.

## 2 Materials

Silver­(I) oxide (Ag_2_O, 99%) having a particle size distribution of 2–12 μm (Figure S1.A) was purchased from abcr GmbH. Prior to use, the oxide was thermally treated to remove the surface carbonate (Ag_2_CO_3_). For this purpose, the powder was heated in a flask at 200 °C for 2 h in the air. This pretreatment temperature was selected based on a preliminary thermal stability study (Figure S1.B). The liquid alcohols were used as purchased and sourced as follows: 1-decanol (≥99.0%) from Sigma-Aldrich, 4-decanol (≥98.0%) from Tokyo Chemical Industry (TCI), and 2,4-pentanediol (≥98%) from Apollo Scientific. Poly­(vinyl alcohol) (PVA) powder (average Mw ∼ 90,000, ≥99% hydrolyzed), oxone (potassium peroxymonosulfate), anhydrous methanol (99.9%), sodium hydrogen carbonate (NaHCO_3_, ≥99.7%), sodium chloride (NaCl, ≥ 99.5%), and molecular sieves (3 Å, 4–8 mesh) were all purchased from Sigma-Aldrich.

## 3 Methods

### 3.1 Thermal Analysis

Differential scanning calorimetry (DSC) measurements were conducted (214 Polyma, Netzsch, Germany) in aluminum crucibles with perforated lids under a constant N_2_ flow of 50 mL/min. The heating rate was fixed at 5 °C/min unless indicated, and sample masses ranged between 4 and 10 mg. For liquid model systems, Ag_2_O and the designated alcohol were mixed directly in the crucible at different mass ratios. In the case of polymer composites, extruded PVA/Ag_2_O samples were ground to millimeter size particles prior to analysis.

### 3.2 Reactive Extrusion and Sample Preparation

Reactive extrusion was carried out using a tabletop twin-screw compounder (MC 15 HT, Xplore, Netherlands). Prior to processing, PVA powder was manually premixed with Ag_2_O at defined mass ratios (Ag_2_O:PVA of 1:1 and 1:10). To minimize oxidative degradation during processing, a constant flow of Ar gas was fed into the hopper of the vertical compounder. The compounding was performed at 240 °C with a screw speed of 50 rpm. The mixture was allowed to homogenize for 2 min to stabilize torque, followed by varying residence times ranging from 30 s to 5 min. For morphological characterization, the extruded samples were first ground to millimeter size particles and then compression-molded into standard films using a hot press (Lindenberg Technics, Switzerland). The pressing protocol lasted for 5 min with pressure increasing incrementally every minute (0, 0.5, 4, 10, and 20 tons), followed by cooling in a cold press under high pressure.

### 3.3 Chemical Analysis in Model Liquids (H_2_O and CO_2_ Determination)

Systematic reduction of Ag_2_O was performed in a labware setup consisting of a round-bottom flask connected to a condenser maintained at −10 °C (Figure S2.A). In a typical experiment, 20 mL of the respective alcohol and 4.8 to 9.9 g of Ag_2_O were introduced into the flask (1:5 mol ratio Ag_2_O:alcohol). The system was purged by executing vacuum/Ar cycles (2 mbar for 20 min, followed by 30 s of Ar backfill) and connected to a gas collector (an inverted graduated cylinder) to monitor the gas evolution. The alcohol mixtures were heated to 150 °C; in all cases, the reaction was held at this temperature until gas evolution ceased. It is worth noting that the system is disproportionately sensitive to temperature changes, and variations in heating rates due to its autocatalytic exothermal nature. H_2_O production was quantified using Karl Fischer Titration (KFT) (906 Titrando Karl Fischer titrator, Metrohm, Switzerland). To ensure total H_2_O recovery, the condenser and the reaction flask were washed with a weighed amount of anhydrous methanol (∼4.5 g for decanol, ∼4.9 g for diol) immediately after the reaction. The mixture was vigorously shaken to homogenize the condensate with the bulk liquid. An aliquot (0.3 to 1.0 mL) was then extracted and injected into the KFT reactor for quantification.

### 3.4 Chemical Synthesis of 4-Hydroxypentan-2-one

It was hypothesized in the study that 2,4-pentanediol is oxidized in two steps. To investigate the selective oxidation of 2,4-pentanediol to 4-hydroxypentan-2-one, dimethyldioxirane (DMDO) was employed as a specific oxidizing agent. DMDO was synthesized as an acetone solution following a standard rotary evaporation protocol.^33^ A slurry consisting of oxone (25 g), sodium hydrogen carbonate (24 g), H_2_O (20 mL), and acetone (30 mL) was prepared in a round-bottom flask. The flask was attached to a rotary evaporator, and the DMDO distillate was collected under reduced pressure (155 mmHg) in a trap cooled by a dry ice/acetone bath (−78 °C). The resulting solution, containing DMDO at a concentration of approximately 0.06 M, was converted with a stoichiometric amount of 2,4-pentanediol.

### 3.5 Chromatography (GC-MS)

Reaction products were identified using gas chromatography–mass spectrometry (GC-MS) (Trace 1300, Thermo Fisher, USA). Compounds were identified using the NIST 2002 MS Library 2.0. Two distinct sampling configurations were employed, liquid phase and headspace analysis. Liquid aliquots were withdrawn directly from the reaction flask under inert conditions to analyze nonvolatile components. Volatile products were analyzed using headspace injections to analyze formed intermediates. Samples were prepared by mixing Ag_2_O and the respective alcohol (1:5 molar ratio) in vials sealed with high-temperature septa. The vials were purged with N_2_ to establish an inert atmosphere. Prior to analysis, the samples were incubated in the autosampler at 150 °C for 10 min, after which the headspace was injected.

### 3.6 Chromatography (GC-TCD)

The quantification of CO_2_ was performed using a gas chromatograph equipped with a thermal conductivity detector (GC-TCD) in a continuous flow configuration (CompactGC 4.0, Brechbühler AG, Switzerland). During the reaction of the 10 g solution (1:5 mol ratio Ag_2_O:alcohol at 100 °C), argon carrier gas was bubbled through the mixture at a constant flow rate of 10 sccm (standard cubic centimeters per minute) (Figure S2.B). The GC-TCD inlet was heated and directly coupled to the reaction flask to prevent the condensation of volatile intermediates. The detector was precalibrated for CO_2_ quantification, with measurements recorded at a sampling intervals of 4 min.

### 3.7 Microscopic and Spectroscopic Characterization

Scanning electron microscopy (SEM) was used to study the morphology of the Ag and Ag_2_O particles, (Hitachi S-4800, Hitachi, Japan) equipped with energy dispersive X-ray (EDX) spectrometry. Samples were prepared by depositing drops of suspended particles (solid phase dispersion or liquid phase supernatant) onto p-type boron-doped silicon wafers or into carbon films. After drying overnight, the samples were coated with a 7 nm gold–palladium layer using a sputter coater (EM ACE600, Leica, Germany). Fourier transform infrared (FTIR) spectroscopy was used to characterize the chemical structure of the polymer matrix (Tensor 27, Bruker, USA). Spectra were collected in attenuated total reflectance (ATR) mode over a range of 650 to 3600 cm^–1^.

The optical absorption properties of the PVA/Ag films were characterized using a UV–vis spectrophotometer (Cary 50, Agilent, USA). Spectra were collected in transmission mode to observe the effect of the embedded Ag.

The concentration of dissolved Ag species was quantified using inductively coupled plasma optical emission spectroscopy (5110 ICP-OES, Agilent, USA). The specific alcohols were stored over activated molecular sieves (30% w/v) for 1 week to achieve low moisture levels.^34^ To separate the dissolved Ag from the solid oxide, a specific filtration protocol was employed: approximately 0.5 g of Ag_2_O was placed in a tube and covered with a layer of dried glass wool. Six mL of the testing liquid were gently added to the tube. The supernatant was then withdrawn through the glass wool layer and subsequently filtered through a 0.45 μm syringe filter to ensure the removal of any larger suspended particulates prior to injection.

### 3.8 Preparation and Testing of PVA/Ag Films via Solution Casting

To prepare the composite films, 60 g of PVA was dissolved in 400 mL of distilled H_2_O at 95 °C (15% w/v concentration). To ensure complete dissolution, the mixture was processed using a high-shear mixer for 10 min. The resulting solution was subsequently cooled to 60 °C, at which point Ag_2_O was introduced at a loading of 0.5% w/w relative to the PVA (Figure S3.A). The suspension was stirred for 15 min without further external heating to ensure dispersion, at which point the solution underwent a color change from gray to yellow, indicating formation of Ag particles (Figure S3.B). Finally, approximately 20 mL of the mixture was dispensed via syringe onto a flat silicon wafer and dried overnight to form the solid film.

To study the optical properties of the PVA/Ag films, they were heated to 120 °C and uniaxially stretched to a draw ratio of 5 (Figure S4). The optical properties of the drawn films were then evaluated using a transmission setup where a yellow LED served as the backlight source, covered by a diffuser to ensure uniform illumination (Figure S5). The light passed through a rotating linear polarizer before illuminating the oriented PVA/Ag film. Digital images were captured using a standard camera with the incident polarization vector oriented in two orthogonal states relative to the film’s drawing direction: perpendicular (electric field orthogonal to the draw direction, resulting in a bright state) and parallel (electric field parallel to the draw direction, resulting in a dark state). The resulting images were processed using ImageJ software to determine the mean pixel intensity of the film in each state. To quantify the optical anisotropy, the darkening percentage was calculated by subtracting the parallel intensity from the perpendicular intensity, dividing by the perpendicular intensity, and multiplying by 100.

The antimicrobial properties of the PVA/Ag films were evaluated using the agar diffusion method against Gram-negative bacteria *Escherichia coli* ATCC 8739. Precultures were grown in Tryptic Soy Broth (TSB) medium overnight at 37 °C with agitation at 160 rpm. For the assay, the bacterial suspension was diluted to an optical density at 600 nm (OD_600_) of approximately 0.1. An aliquot of 100 μL of the suspension was spread onto Tryptic Soy Yeast (TSY) agar plates and allowed to dry for 30 min. The PVA/Ag composite samples were then placed in triplicate onto the inoculated agar surface. The plates were incubated overnight at 37 °C to allow for bacterial growth and the diffusion of antimicrobial agents. Finally, the zone of inhibition surrounding each sample was imaged and quantified using an automatic colony counter (Scan 300, Interscience, France).

## 4 Results and Discussion

### 4.1 PVA/Ag_2_O Polymer Mobile Phase

Ag_2_O is known to undergo thermal reduction to metallic Ag under a N_2_ atmosphere, typically concluding at a temperature around 407 °C (Figure 1). Analysis of neat Ag_2_O revealed an initial mass loss at 196 °C, which was attributed to the decomposition of surface silver carbonate (Ag_2_CO_3_) contaminants into Ag_2_O (Figure S1.B). Consequently, a thermal pretreatment of the Ag_2_O was necessary to reproducibly eliminate surface carbonates prior to use.

**Figure 1:**
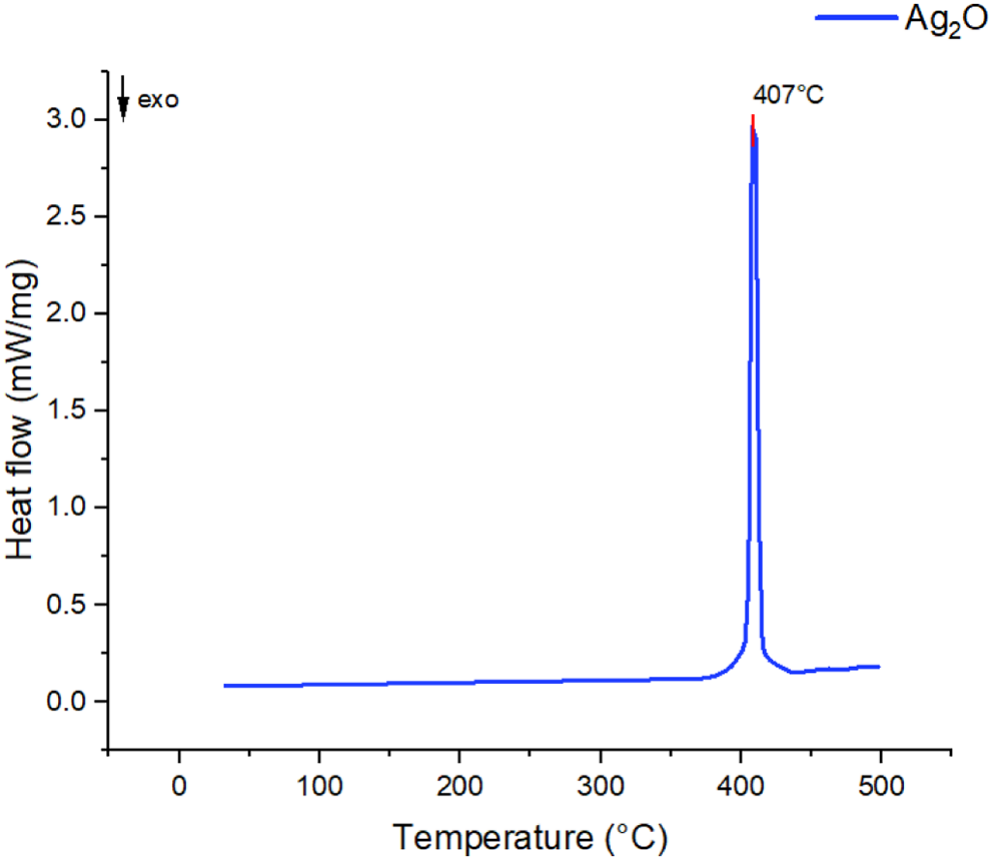
DSC of Ag_2_O at a 10 °C/min heating rate under N_2_ flow. An endothermal peak, attributed to the decomposition of Ag_2_O, appears at 407 °C.

To elucidate the influence of the polymer matrix on the reduction of Ag_2_O, the thermal behavior of PVA/Ag_2_O blends was first analyzed using DSC under a N_2_ atmosphere. For comparison, the thermal profiles of neat PVA, neat PE, and a PE/Ag_2_O blend were also evaluated (Figure 2). PE was selected as a nonpolar control because, in contrast to PVA, it lacks the hydroxyl functional groups. As established previously,^12^ the reduction of Ag_2_O in PE proceeds via a radical mechanism, initiated by the formation of polymer radicals at elevated temperatures.

**Figure 2:**
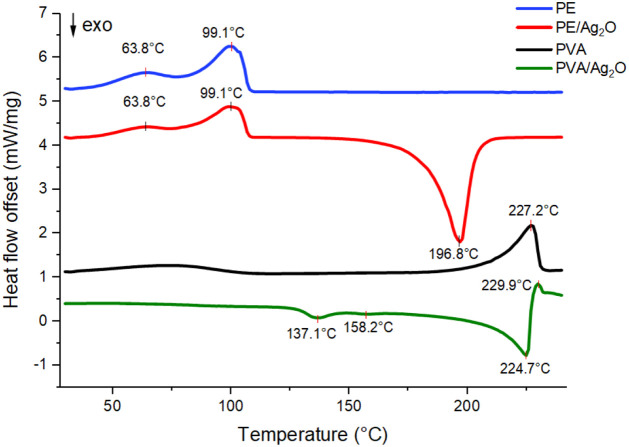
DSC thermograms of neat PE, PE/Ag_2_O blend (1:1 w/w), neat PVA, and PVA/Ag_2_O blend (1:1 w/w), obtained at a heating rate of 5 °C/min under N_2_ flow.

The thermal behavior of the PE control group was analyzed first (Figure 2). Neat PE displayed a first melting endothermal peak at 63.8 °C corresponding to the melting of secondary crystallites,^35^ followed by the melting of the main crystalline phase at 99.1 °C. The corresponding Ag_2_O/PE dry mixture (1:1 w/w) exhibited besides the two endothermal peaks of PE also a single exothermic peak at 196.8 °C, which corresponds to the reduction of the Ag_2_O.

The DSC thermogram of neat PVA powder reference exhibited a broad endothermic peak ranging from room temperature to 100 °C, attributed to the evaporation of absorbed moisture, consistent with the hygroscopic nature of PVA (Figure 2). A distinct endothermic peak corresponding to the melting of the polymer was observed at 227.2 °C. The thermal profile of the PVA/Ag_2_O mixture (1:1 w/w) revealed this endothermic peak only slightly; instead three distinct exothermic peaks emerged. Two minor peaks were detected at 137.1 and 158.2 °C, followed by a pronounced peak at 224.7 °C, which was attributed to the reduction of Ag_2_O. A deflection point was observed on the shoulder of this main peak at approximately 229.9 °C, suggesting that the endothermic melting transition of PVA was overlapped by the larger exothermic reduction. The retardation of the primary reduction peak to the polymer’s melting temperature suggests that the bulk reduction of Ag_2_O is initiated only after the PVA matrix reaches collective fluidity.

Comparing the two polymers reveals that the main reduction in PE (196.8 °C) occurs at a lower temperature than the main reduction in PVA (224.7 °C). This is counterintuitive, as the hydroxyl functional groups in PVA are more susceptible to participate in redox reactions compared to the saturated hydrocarbon chains of PE, which melt at a significantly lower temperature (99.1 °C) than PVA (227.2 °C). Again, this supports the idea of the critical role of the polymer fluidity to allow reactions between alcohol groups and Ag_2_O. In the solid state, the interaction between the salt and the polymer is consequently localized to the Ag/PVA interface. Similarly, it is suggested that the small peaks at 137.1 and 158.2 °C would correspond to localized reduction of Ag^+^ at the solid PVA interface.

To validate this mobility-limited hypothesis, PVA was dissolved in H_2_O (15% w/v concentration) to mobilize the chains at lower temperatures; this resulted in a reduction of Ag^+^ at 60 °C (Figure S3). Such low reduction temperatures in dissolved PVA suggests that the minority peaks at 137.1 and 158.2 °C are also local reduction reactions that are enabled by a local chain mobilization.

Furthermore, we note that H_2_O acts solely as a solvent and is not the reducing agent for Ag_2_O, as it is chemically unable to donate electrons or accept oxygen under these conditions (Figure S6). This implies that molecular mobility to install proximity between Ag_2_O and the alcohol groups of PVA is a key factor for the redox reaction to proceed.

Since the DSC analysis highlighted the role of polymer collective mobility in initiating the reduction, the molecular weight distribution (MWD) of the PVA was further examined; namely, any low MW fractions could offer enhanced mobility at lower temperatures and thus provide a possible explanation for the observation of small peaks below the bulk melting temperature. The measured MWD of the PVA used in this study (99% hydrolyzed, nominal Mw 90 000 g/mol, designated as “PVA powder”) is presented in Figure 3. The chromatogram, plotted as the differential weight fraction (dW/dLogM) against the molecular weight (*M*_w_), indeed reveals a bimodal distribution. The profile is characterized by a dominant population with a peak molecular weight (M_p_) of approximately 90 500 g/mol, along with a minor fraction in the low molecular weight range of 3000–5000 g/mol. This minor fraction could be attributed to residual oligomers, which should exhibit higher chain mobility compared to the bulk polymer.

**Figure 3:**
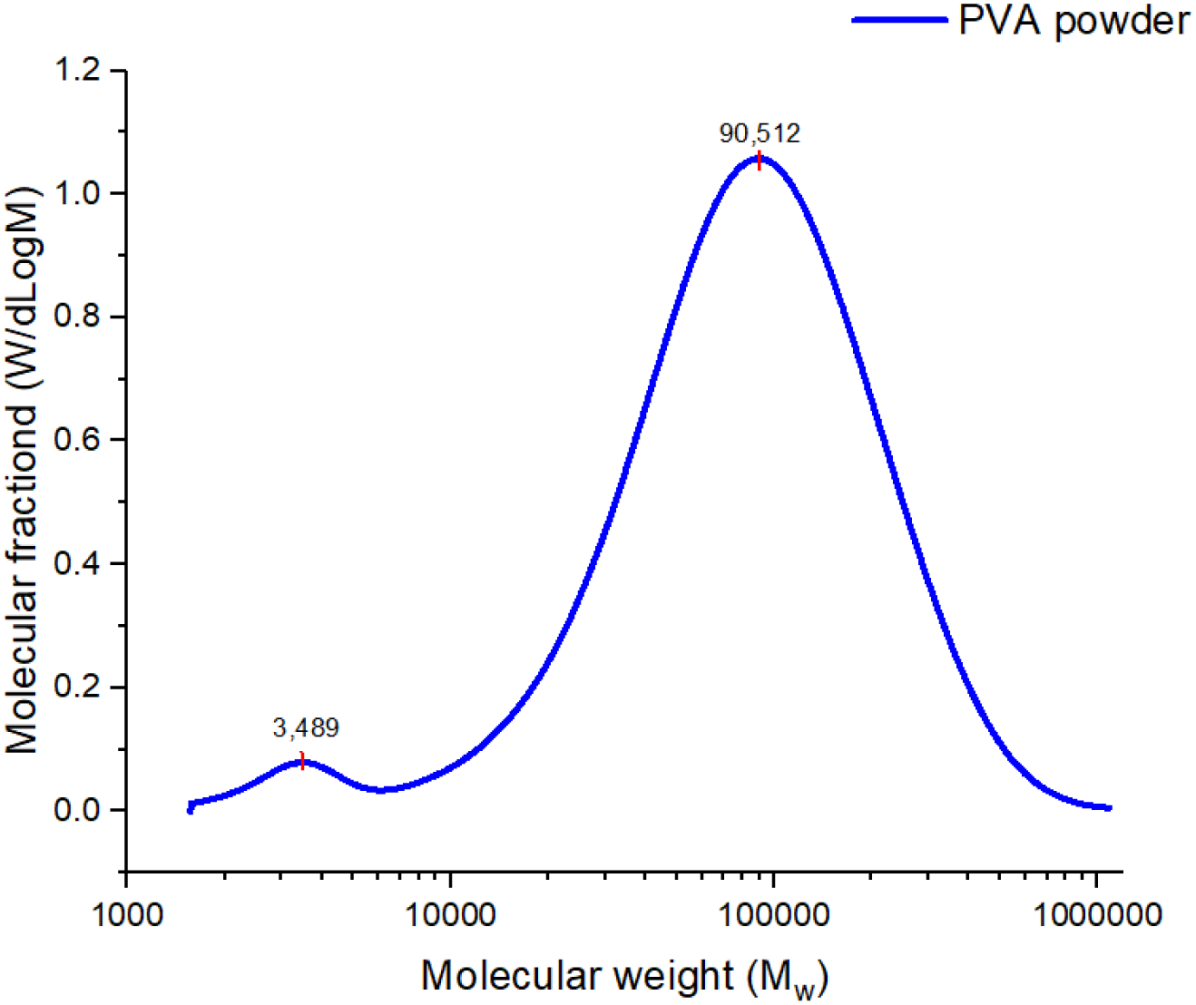
MWD of the neat PVA powder (99.9% hydrolyzed). The chromatogram exhibits a bimodal distribution characterized by a dominant population with a peak molecular weight (M_p_) of 90 500 g/mol and a minor low-molecular-weight fraction at 3490 g/mol.

According to literature, the presence of H_2_O could also influence chain mobility, as H_2_O is a known plasticizer for PVA.^36,37^ H_2_O exists within the polymer matrix in different states, including bound water which can lower the melting temperature. A significant melting temperature decrease often typically relates to low molecular weight PVA (∼2000 g/mol).^36^ In this study, the addition of 50% w/w H_2_O to the high molecular weight PVA powder did not result in a significant reduction of the melt temperature of the PVA (Figure S7). This suggests that H_2_O-induced plasticization is not the primary driver for molecular mobility. Instead, the early reduction events observed in Figure 2 are thus likely facilitated by the enhanced mobility of the low molecular weight oligomer fraction identified in the MWD analysis. Indeed, such early endothermal peaks before the main melt temperature peak have been observed in PVA by several studies.^38–40^ In summary, H_2_O is unreactive and does not depress the melting temperature of high-molecular-weight PVA. However, a highly mobile aqueous system (PVA dissolved in water with Ag_2_O) enables reduction at just 60 °C. This concurs with the idea that the chemical interactions are fundamentally limited by physical diffusion and chain mobility.

To further validate the hypothesis that localized polymer chain mobility limits the reduction reaction, we conducted a series of heat-up experiments with different Ag_2_O loadings. The idea is to determine how much Ag_2_O can be reduced by local mobility below the melting temperature. DSC analysis was performed on a physical mix with either a low Ag_2_O loading (Ag_2_O/PVA, 1:10 w/w), or, a higher loading (1:1 w/w) as well as a reference with neat PVA (Figure 4). The thermogram of the 1:10 mixture revealed no significant exothermic reduction peak near the polymer’s melting transition; however, the two smaller peaks are still present. The addition of such smaller amounts of Ag_2_O indeed resulted in a significant increase in the degree of crystallinity (*X*_c_), rising from 38.7% in neat PVA to 53.8% in the composite, taken ${\Delta H}_{f}^{0}$ as 138.6 J/g^41^ (Table 1). This sharp increase, accompanied by a 39% rise in melting enthalpy is in line with the idea that the Ag_2_O particles act as nucleating agents, promoting the ordering of PVA chain.

**Figure 4:**
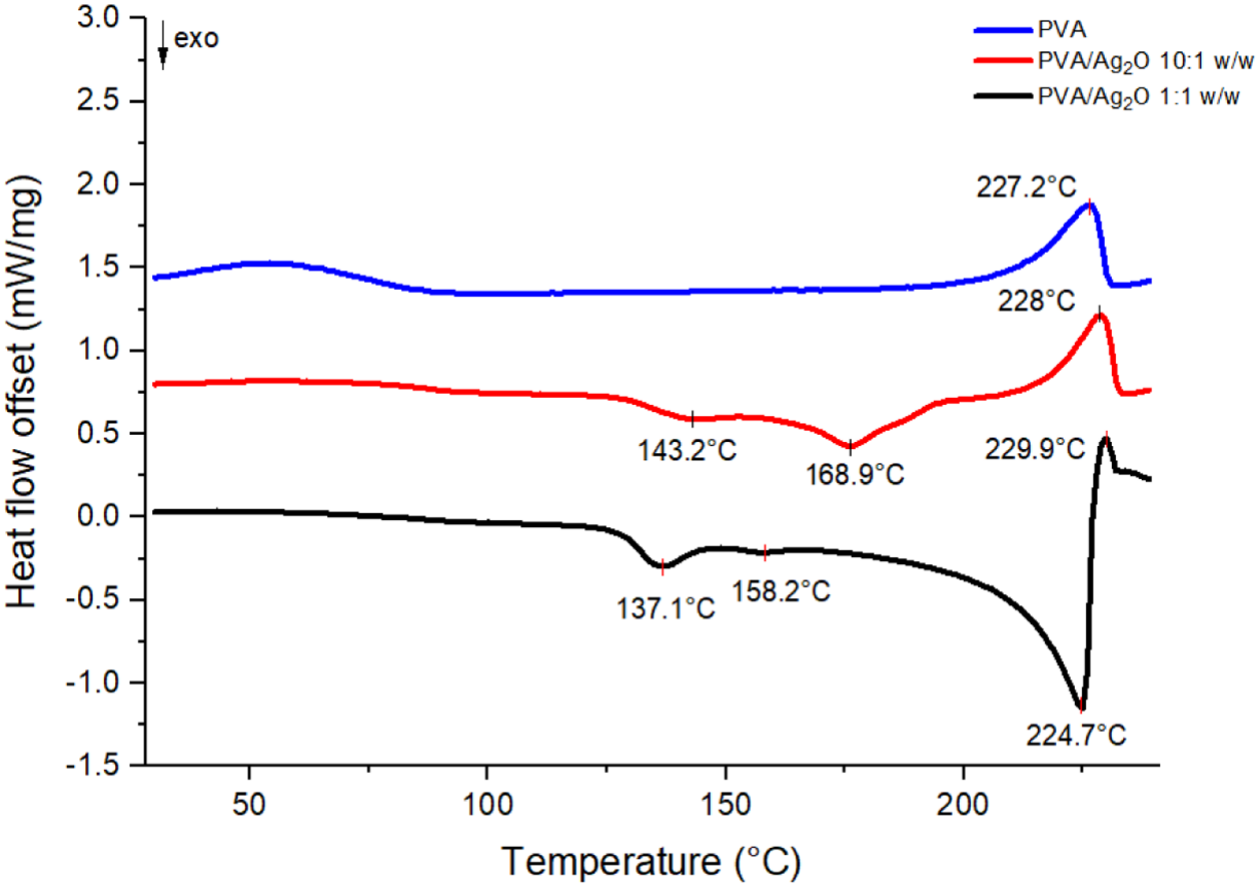
Comparative DSC thermograms of neat PVA, PVA/Ag_2_O (10:1 w/w) blend, and PVA/Ag_2_O (1:1 w/w) blend. The 10:1 blend, containing a higher proportion of polymer matrix, lacks the distinct exothermic reduction peaks during melting, as observed in the higher loaded 1:1 blend.

**Table 1:** Thermal Characterization of Neat PVA and PVA/Ag_2_O (10:1 W/W) Physical Blend

Sample	ΔH_m_ (J/g)	T_m_ (**°**C)	X_c_ (%)
Neat PVA	53.6	226.8	38.7
PVA/Ag_2_O (10:1)	74.6	228.0	53.8

The absence of a major reduction peak at the melting temperature in the sample with lower Ag_2_O content (Ag_2_O/PVA, 1:10 w/w) suggests that a small amount of Ag_2_O can be completely reduced at lower temperatures.

The DSC analysis performed on a high loading system (Ag_2_O/PVA, 1:1 w/w) still showed the two minor low-temperature peaks. Cooling to room temperature before reaching the main reduction peak followed by reheating, resulted in minor peaks being absent on the second pass, and only the primary reduction peak near the melting temperature was observed (Figure 5). This disappearance reveals that the initial peaks belong to an irreversible reaction; the working hypothesis is that local reduction of Ag_2_O occurs with mobile hydroxyl groups, at least partially associated with low molecular weight fractions in the polymer.

**Figure 5:**
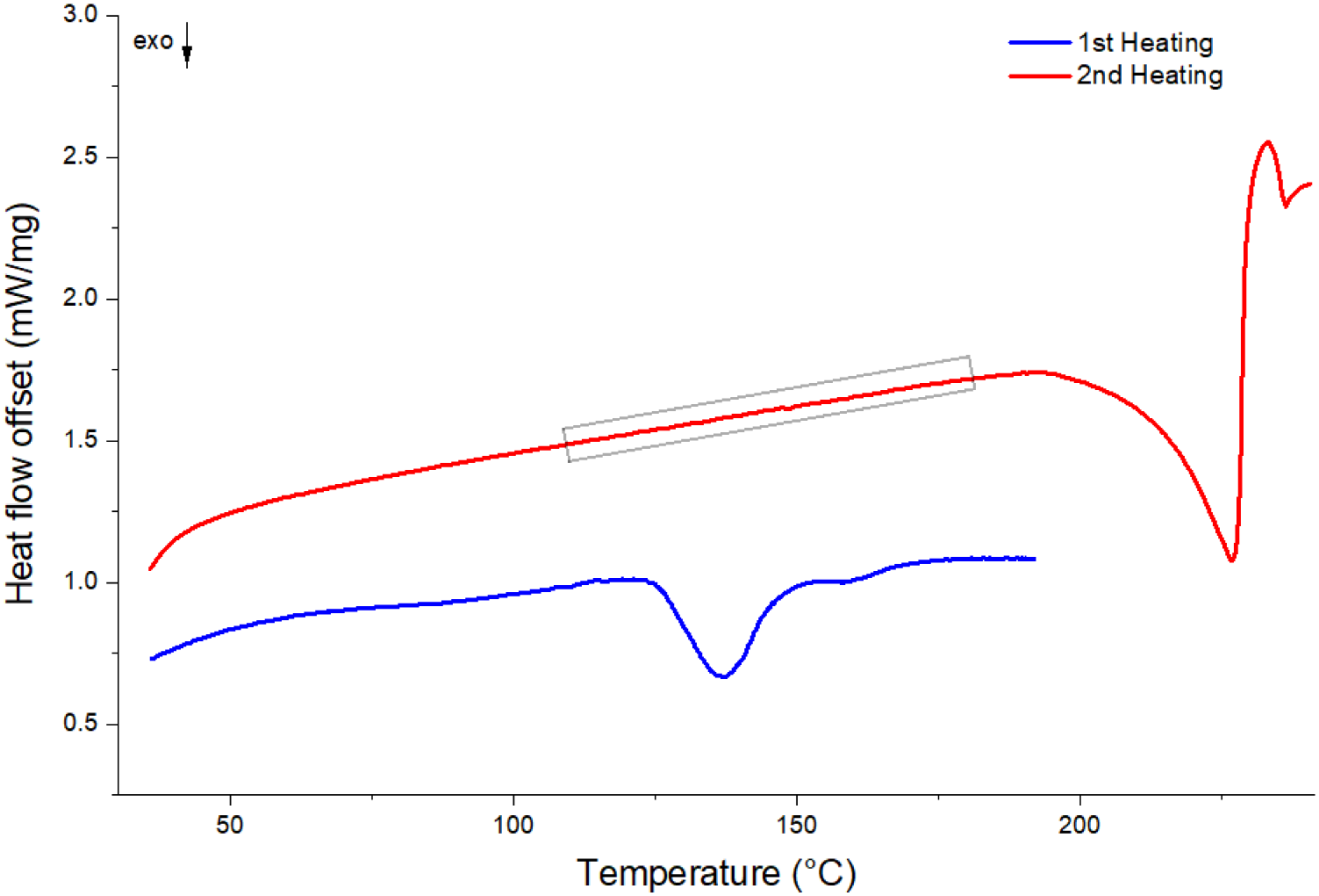
DSC thermograms of PVA/Ag_2_O (1:1 w/w) at two heating cycles, obtained at a heating rate of 5 °C/min under N_2_ flow.

### 4.2 Ag_2_O/PVA in Polymer Melt (Polymer Mobile Phase)

To the best of our knowledge, the melt extrusion of PVA with Ag_2_O has not been previously reported, although several studies have investigated the use of other Ag salts, such as silver nitrate (AgNO_3_) with PVA.^42^ To study the melt extrusion scenario, neat PVA and the PVA/Ag_2_O blend (10:1 w/w) were melt-compounded at 240 °C for varying durations (30 s, 2 min, and 5 min) and extruded into strands (Figure 6). Reduction of the Ag_2_O was observed to proceed immediately at these processing temperatures. Even at the shortest residence time (30 s), the extrudate exhibited a characteristic dark brown color and an abundant formation of voids. Postprocessing DSC analysis of the extruded strands revealed no exothermic reduction peaks prior to the melting transition (Figure 7), confirming that the Ag_2_O was completely reduced to metallic Ag during the melt compounding step. The melt temperature peak of the PVA/Ag_2_O strands (Figure 7) are slightly shifted to 228–229 °C but it is unclear if this shift has to be considered as significant as such small shifts might be caused e.g., by differences in the water content of the PVA.

**Figure 6:**
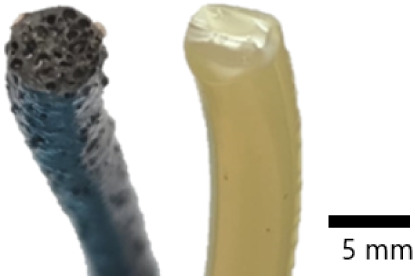
Digital images of extruded strands of neat PVA (right) and PVA/Ag_2_O (left) (10:1) compounded at 240 °C, showing color change and void formation in the composite.

**Figure 7:**
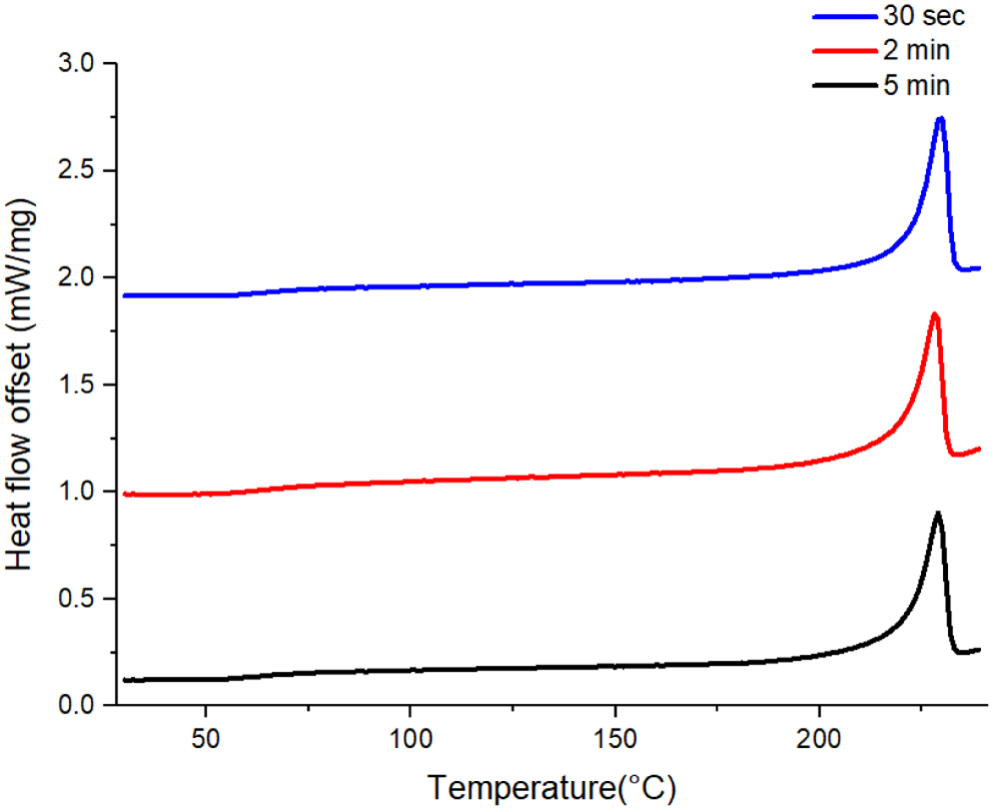
DSC thermograms of the extruded strands, showing the absence of reduction peaks, indicating complete conversion of Ag_2_O to Ag during processing.

Attempts to minimize thermal reduction by lowering the extrusion temperature were constrained by the thermal properties of PVA. While extruding the polymer exhibits a narrow processing window, indeed acting as a rigid solid just few degrees below its melting temperature. Unlike the neat PVA strands, the Ag-composite extrudates contained macroscopic voids (millimeter scale). These voids result from the expected gas evolution (likely H_2_O and CO_2_) due to the oxidation of the PVA matrix by the Ag_2_O at these elevated temperatures (Figure 6). Quantification of these gases during extrusion was not available, thus the model system approach was adopted.

To determine the Ag particle morphology, the extruded strands were milled into powder and compression molded into thin plates (Figure 8). A clear contrast was thus obtained between the samples; namely, the Ag loaded plates exhibited a deep brown coloration, consistent with the formation of metallic Ag nanoparticles as suggested by others.^43–45^ In contrast, the neat PVA control plates displayed a distinct yellow discoloration.

**Figure 8:**
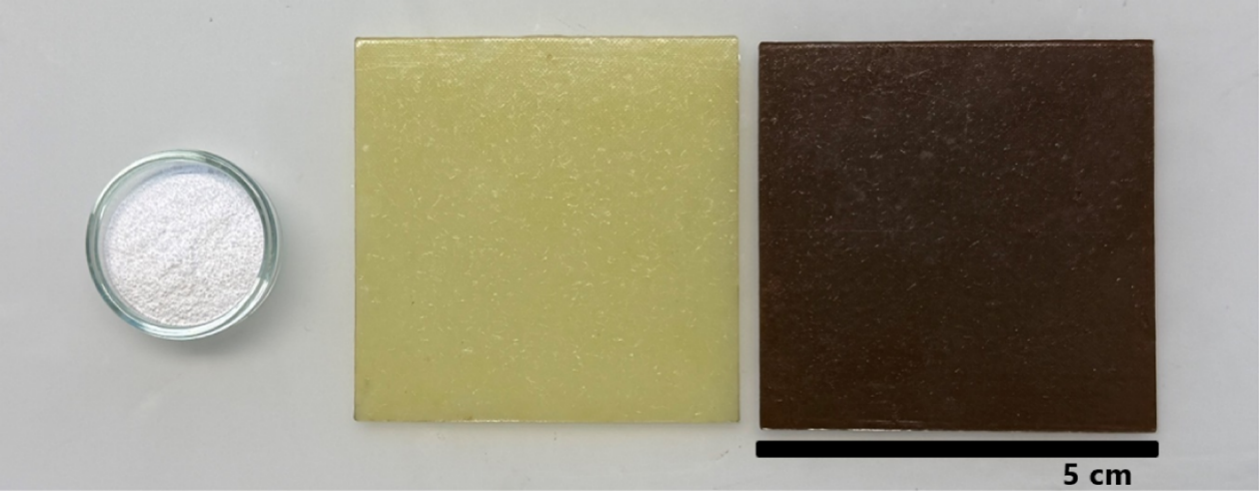
Digital image of neat PVA powder (white color), PVA compounded for 2 min (pale yellow plate), PVA/Ag_2_O (10:1 w/w), showing the characteristic dark brown color of Ag particles.

This yellowing exhibits the challenge of processing PVA, which is characterized by a narrow thermal processing window. Unlike thermally stable thermoplastics such as PE, the onset of thermal degradation for PVA (∼230–250 °C) occurs in the proximity to its melting temperature (227 °C). Consequently, compounding at 240 °C exposes the polymer to thermal stress sufficient to initiate degradation. This mechanism is analogous to the side-group elimination observed in poly­(vinyl chloride) (PVC). Indeed, the color change of PVA is attributed to the thermal dehydration of the polymer backbone. The elimination of pendant hydroxyl groups, leaving as H_2_O, leads to the formation of conjugated carbon–carbon double bonds (polyene sequences).^46^ As the length of these conjugated systems increases, they are known to act as chromophores that absorb light in the blue region of the visible spectrum, resulting in the yellow appearance observed in the neat polymer after compounding.^47,48^

To investigate the formation of oxidation products, FTIR spectroscopy was performed on neat PVA, PVA compounded for 2 min, and PVA compounded for 2 min with Ag_2_O (1:10 w/w) (Figure 9). The FTIR spectrum of the neat PVA powder exhibited a broad peak at 3277 cm^–1^, attributed to O–H stretching vibrations. The broadening of this band is characteristic of strong hydrogen bonding between hydroxyl groups and hydroxyl groups and water. This feature was observed across all three samples.

**Figure 9:**
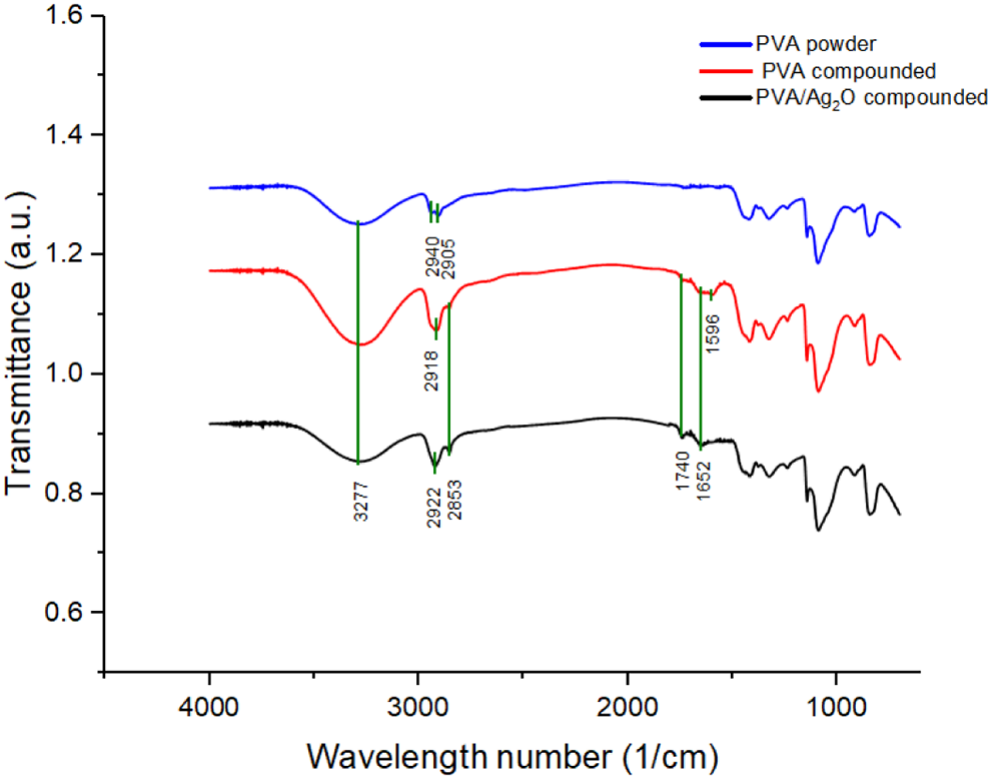
FTIR spectra of neat PVA, PVA compounded for 2 min, and PVA compounded with Ag_2_O (10:1 w/w). The appearance of bands at 1740 cm^–1^ (C=O) and 1652 cm^–1^ (C=C) in the compounded samples indicates thermal oxidation and dehydration. The increased intensity of these peaks in the presence of Ag_2_O suggests enhanced degradation due to the presence of the oxidant.

Additionally, the neat PVA spectrum showed two narrow peaks at 2940 cm^–1^ and 2905 cm^–1^, assigned to the asymmetric and symmetric C–H stretching vibration of the alkyl groups, respectively.

Significant spectral changes were observed after compounding. In the compounded PVA heat pressed sample, the C–H stretching vibrations red-shifted, with dominant peaks moving to 2918 cm^–1^ and 2853 cm^–1^. These shifts suggest a change in the polymer backbone structure. While the small peak at 1740 cm^–1^ is attributed carbonyl (C=O) stretching vibrations, a peak at 1652 cm^–1^ is in the region of alkene (C=C) stretching vibrations, respectively. These signals indicate that the polymer underwent both oxidation and dehydration during processing.^46^ A new absorption band at 1569 cm^–1^ might be attributed to the stretching of conjugated C=C bonds (polyenes) or conjugated carbonyl groups. This is in line with the propagation of degradation along the polymer backbone and explains the visible color shift to pale yellow observed in Figure 8, highlighting the sensitivity of PVA to thermal processing.

The spectrum of PVA compounded with Ag_2_O was dominated by intense peaks at 1740 cm^–1^ and 1652 cm^–1^. The sharpness and intensity of these signals suggest that the oxygen supplied by the Ag_2_O promoted carbonyl formation facilitated by hydrogen abstraction on the polymer.

Having characterized the chemical modification of the polymer matrix via FTIR spectroscopy, it is also interesting to track the morphological evolution and elemental composition of the in situ generated Ag phase enclosed within the extrudates. The reactive compounding of Ag_2_O with PVA led to the formation of metallic Ag particles (Figure 10), as confirmed by EDX analysis. The atomic ratio of carbon to oxygen was found to be ∼2:1, with a mass ratio of ∼1.5:1, which is directly consistent with the stoichiometry of the PVA matrix. This confirms the oxygen from Ag_2_O has been removed and reduction occurred within a 30 s compounding window. Morphologically, SEM micrographs of the PVA/Ag_2_O samples reveal that the metallic phase consists of roughly spherical nanoparticles with an estimated diameter between 100 and 300 nm. However, these particles are largely clustered into irregular agglomerates ranging from approximately 0.5 to 3.0 μm. This clustering reflects the high surface free energy typical of metallic nanomaterials. It is interesting to note that the size of these agglomerates is comparable to the initial Ag_2_O particle size (2–5 μm), suggesting that the particles underwent in situ reduction and volume contraction without achieving significant dispersive mixing with the polymer matrix. This lack of distribution can be attributed to the rapid reaction kinetics at the processing temperature of 240 °C and the inability of Ag species to dissolve in the bulk polymer melt. Furthermore, the high processing temperature likely contributes to this agglomeration. While the alcohol can reduce Ag_2_O at much lower temperatures, the reaction is restricted by the solid polymer matrix. Upon melting, rapid reduction occurs immediately. The low melt viscosity combined with the immediate reduction of Ag_2_O prevented the shear forces from breaking up the clusters by melt shear forces. As will be further illustrated in the application section, the reduction of Ag_2_O in contact with a convectively mobile PVA phase is thermodynamically favorable and can initiate at the particle surface at temperatures as low as 60 °C. We can thus adopt the plausible hypothesis that the extrusion process successfully facilitated the rapid, in situ synthesis of Ag nanoparticles in a solid–solid transformation process.

**Figure 10:**
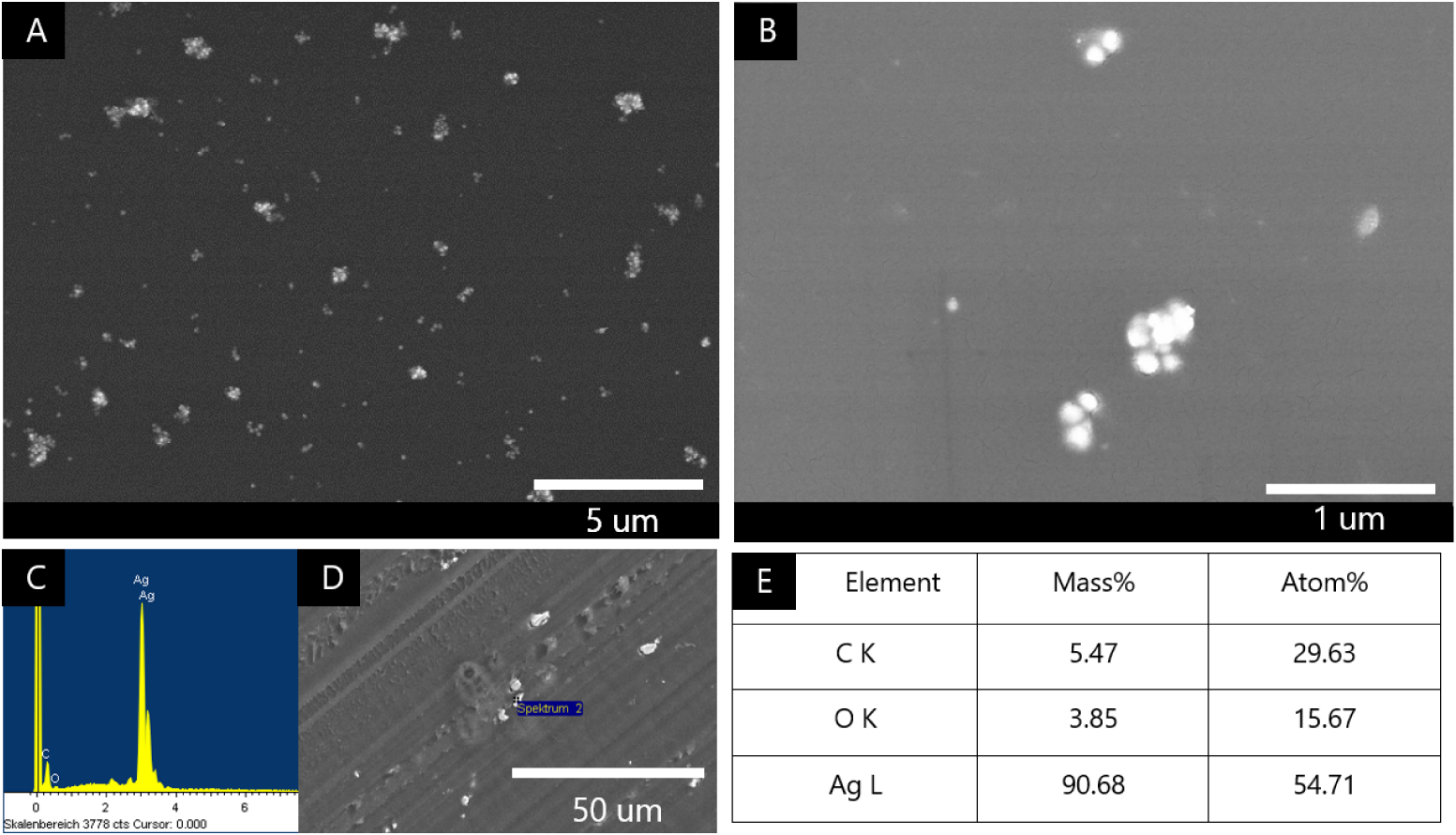
SEM micrographs of PVA/Ag_2_O (10:1 w/w) blends compounded at 240 °C after 30 s and large magnification. (A) shows the general distribution of the clusters, while (B) presents a higher magnification view of the particle morphology. (C, D, E) The corresponding EDX analysis confirms the elemental composition of the metallic Ag phase.

### 4.3 Ag_2_O Reduction in Model Liquids (Model Liquid Mobile Phase)

The polymer extrusion process introduces a complex mobility dynamics due to the polymer chains described above, significantly influence the reduction of Ag_2_O. A possible complication is the mechano-thermal coupling during extrusion, where shear stresses generated in the barrel can accelerate reaction kinetics and induce viscous heating, resulting in localized high temperature zones. Furthermore, despite efforts to maintain an inert environment, trace atmospheric oxygen may alter the reduction pathway in an extrusion process.

To decouple these processing factors from the fundamental chemical reaction, and to overcome the narrow processing window and limited molecular mobility of PVA, a model system was employed that remained liquid at ambient temperature. Namely, 2,4-pentanediol was selected as a compound that is chemically similar to PVA. Additionally, 1-decanol and 4-decanol were employed to investigate the specific reactivity differences between terminal hydroxyl groups and internal hydroxyl groups.

To investigate the reduction kinetics of Ag_2_O submerged in the chosen model liquids, DSC in closed crucibles was performed using a 1:1 (w/w) ratio of Ag_2_O to liquid. The resulting thermograms (Figure 11) reveal distinct reduction profiles for primary and secondary alcohols. 1-decanol exhibited a relatively sharp exothermic reduction peak centered at 161.5 °C, whereas 4-decanol displayed a peak at a significantly lower temperature of 153.2 °C.

**Figure 11:**
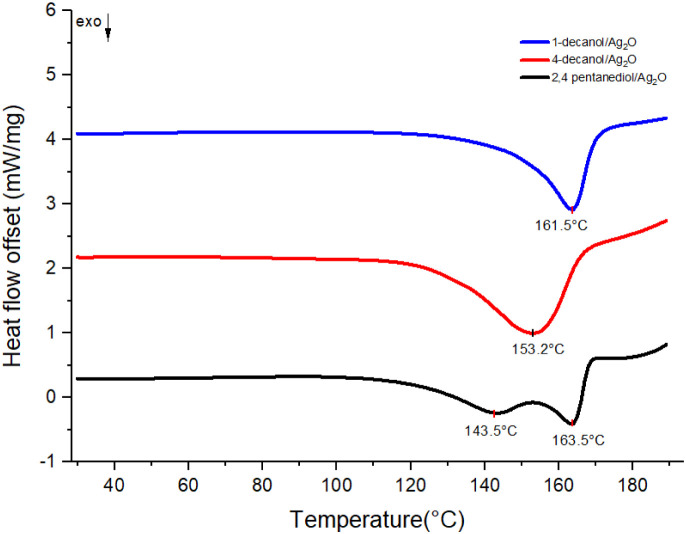
DSC thermograms of Ag_2_O mixed with model liquids (1:1 w/w). The curves compare the reduction behavior of a primary alcohol (1-decanol, peak at 161.5 °C), a secondary alcohol (4-decanol, peak at 153.2 °C), and a diol (2,4-pentanediol, peaks at 143.5 and 163.5 °C).

While 4-decanol initiates the reduction of Ag_2_O at a lower temperature, it exhibits a much broader reaction window (onset-to-offset temperature) compared to 1-decanol. This contrasting thermal behavior is likely attributed to the steric accessibility of −OH groups at the interface to Ag_2_O. The lower onset temperature for 4-decanol concurs with the lower bond dissociation energy (BDE) of the secondary α-C–H bond (∼91 kcal/mol, based on propan-2-ol analogues)^49^ compared to that of the primary α-C–H bond (∼95 kcal/mol, based on 1-propanol).^49^ As C–H bond scission is the rate-limiting step, a weaker secondary bond facilitates easier hydrogen abstraction. Furthermore, the transition state (radical or cationic character) of secondary alcohols are in general better stabilized better than in primary alcohols.^50^

However, despite this thermodynamic advantage facilitating early initiation, the reaction window is significantly broadened possibly due to physisorption-related steric limitations at the surface. Unlike the terminal hydroxyl group of 1-decanol, the internal hydroxyl group of 4-decanol faces geometric restrictions on both sides when approaching the solid Ag_2_O surface. Additionally, the desorption of the bulky ketone product (4-decanone) is kinetically slower than that of the linear aldehyde. This likely leads to a higher surface population of physiosorbed molecules with kinetic limitations, practically extending the thermal range required to achieve full conversion. In contrast to the monoalcohols, the model diol, 2,4-pentanediol, exhibited a bimodal reduction profile in DSC diagrams with distinct exothermic peaks at 143.5 and 163.5 °C (Figure 11). In a cumulative event, the total reaction window is broadened, similar to that of 4-decanol. Notably, this double peak behavior mirrors the thermal reduction characteristics observed in the PVA/Ag_2_O polymer (Figures 2 and 4), suggesting that 2,4-pentanediol serves as an accurate model for the polymer backbone. We hypothesized that these two peaks correspond to a stepwise, local oxidation mechanism.

To identify the intermediate species responsible for the second peak, a DSC experiment was conducted and stopped at a temperature of 150 °C; exactly between the two thermal events and then immediately quenched. The resulting reaction mixture was analyzed via GC-MS (Figure 12.A). The analysis revealed two primary oxidation products: acetic acid (trace, relative area 0.09%) and 4-hydroxypentan-2-one (major intermediate, relative area 7.8%). Control experiments revealed that acetic acid reduces Ag_2_O instantaneously at room temperature (Figure S8); therefore, this reaction cannot be involved in the high temperature peak at 163.5 °C.

**Figure 12:**
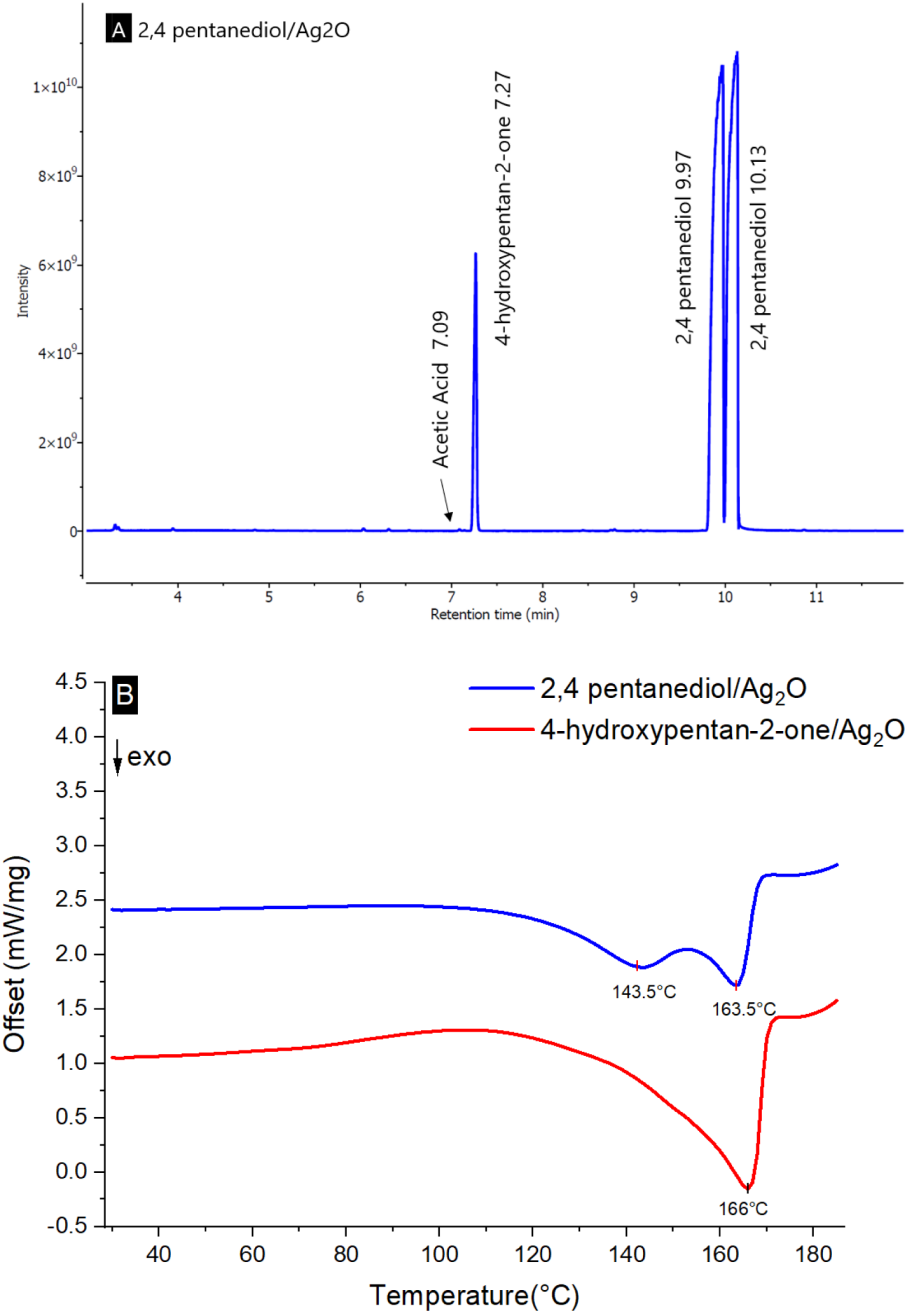
(A) GC-MS chromatograph of liquid phase sample of a DSC experiment, which was conducted at 150 °C then quenched. (B) DSC reaction of 4-hydroxypentan-2-one and 2,4-pentanediol with Ag_2_O.

To understand more about the possible role of the second −OH group in the second minor peak in the DSC at 163.5 °C, 4-hydroxypentan-2-one was independently synthesized. Therefore, dimethyldioxirane (DMDO) was generated from the reaction of oxone with acetone. This solution was utilized for the selective mono-oxidation of 2,4-pentanediol to yield 4-hydroxypentan-2-one, the structure of which was confirmed by GC-MS (Figure S9).

When the synthesized 4-hydroxypentan-2-one reacted with Ag_2_O, the DSC thermogram showed a single reduction peak at 166 °C (Figure 12.B). This value closely corresponds to the second reduction peak observed in the DSC thermogram of 2,4-pentanediol (163.5 °C). These results strongly suggest a sequential reaction pathway, the diol is partially oxidized to the hydroxy ketone during the first thermal event, and this intermediate then reacts with Ag_2_O at slightly higher temperatures.

### 4.4 Ag_2_O/Model Liquids Volatile Study (Model Liquid Mobile Phase)

To understand how the reaction of Ag_2_O proceeds, it is necessary to investigate the reaction products. The chemical evolution of gaseous products during the reduction of Ag_2_O in model alcohols (1:5 mol/mol) was monitored at two distinct temperature settings, each utilizing dedicated setup (Figure S2). Reaction kinetics were studied via GC-TCD (gas chromatography with thermal conductivity detector) at 100 °C by the gas evolution rate (Figure 13). Using GC-TCD, only CO_2_ was measured. Complementary gas displacement volume and Karl Fischer titration (KFT) analyses were conducted in a dedicated labware setup at 150 °C, where both CO_2_ and H_2_O were measured. This temperature was selected to mimic realistic extrusion conditions and to limit the Ag nanoparticle catalytic activity. Across all model liquids, the reaction demonstrated high levels of degradation products resulting from oxidation of the alcohols; the gaseous products consisted exclusively of CO_2_ and H_2_O, and neither O_2_ nor CO was detected.

**Figure 13:**
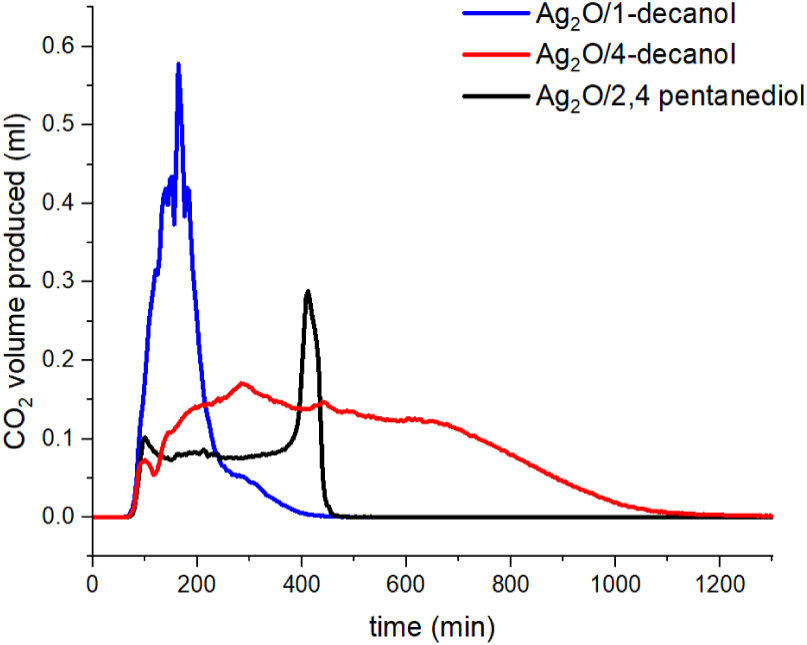
CO_2_ evolution profiles at 100 °C for various model alcohols, measured via GC-TCD using an Ag_2_O:alcohol ratio of 1:5 mol/mol. The total volume of evolved CO_2_ was determined by integrating the area under each curve. Assuming a stoichiometric requirement of two moles of Ag_2_O per mole of generated CO_2_, these volumes correspond to an equivalent Ag_2_O conversion of 21.7% (1-decanol), 43.4% (4-decanol), and 15.1% (2,4-pentanediol) relative to the initial oxide mass.

For 1-decanol at 100 °C (Figure 13), CO_2_ evolution peaked after 170 min and ceased after approximately 400 min. The reaction yielded a total CO_2_ volume of 12.6 mL, as determined by GC-TCD, using 1.2 g of Ag_2_O. Assuming a stoichiometric requirement where two moles of Ag_2_O provide one mole of CO_2_, the theoretical maximum volume is 58 mL. Therefore, the measured 12.6 mL corresponds to an equivalent Ag_2_O conversion of 21.7% (Table 2). The initial acceleration in CO_2_ production of 1-decanol is the highest of all alcohols (Figure 13). However, increasing the temperature to 150 °C drastically altered the product stoichiometry, the CO_2_ conversion rose to 49.1% (Table 2), accompanied by a 49.2% equivalent conversion based on H_2_O production, where one mole of Ag_2_O provides equivalent one mole of H_2_O. This upward trend aligns with a report in the literature on the reaction of Ag_2_O with 1-tetradecanol, where equivalent Ag_2_O conversions of ∼61% (calculated from CO_2_) and ∼35% (calculated from H_2_O) were observed at significantly higher temperatures (∼200 °C).^51^ The progression from 21% (100 °C) to 49% (150 °C) might be due to two reactions, dehydrogenation and oxidation which are both temperature-dependent; a higher temperature would in this case favor CO_2_ generation over dehydrogenation.

**Table 2:** Measured CO_2_ and H_2_O Using Two Methods, GC-TCD and Labware Setup^a^

Equivalent Ag_2_O conversion (%)	1-decanol	4-decanol	2,4-pentanediol
calculated from CO_2_ (100 °C)	21.7%	43.4%	15.1%
calculated from CO_2_ (150 °C)	49.1%	46.8%	33.3%
calculated from H_2_O (150 °C)	49.2%	50.3%	92.4%

a The “Equivalent Ag_2_O conversion” represents how much of the oxygen from the starting Ag_2_O resulted in the measured CO_2_ or H_2_O.

The reaction with 4-decanol at 100 °C exhibited a beginning of the CO_2_ production at same time as 1-decanol but then the CO_2_ evolution became sluggish and the maximum rate was reached only at 300 min and CO_2_ evolution only ended at about 1100 min, by far longer than with the other alcohols (about 400–450 min) (Figure 13). Among the tested alcohols, 4-decanol showed the slowest effective reaction rate and the highest conversion, with a 43% equivalent Ag_2_O conversion to CO_2_ at 100 °C. This prolonged reaction time frame aligns with the thermal study findings and might be due to steric effects. At 150 °C, CO_2_ conversion increased only marginally to 46.8%, accompanied by an H_2_O conversion of 50.3% (Table 2). The consistency of these values across temperatures suggests that the oxidative reaction for secondary alcohols is robust and less dependent on thermal activation, in contrast to 1-decanol.

Finally, the reaction of 2,4-pentanediol with Ag_2_O at 100 °C initiated CO_2_ evolution at a time similar to the other alcohols, but rapidly reached a plateau that persisted for almost 300 min (Figure 13). Subsequently, CO_2_ evolution increased rapidly, reaching a maximum rate around 400 min. Under these isothermal conditions at 100 °C, the total measured CO_2_ corresponded to an equivalent Ag_2_O conversion of only 15.1%. This two-stage behavior aligns with observations from the DSC thermal study, suggesting at least two site-specific reaction steps. Raising the reaction temperature to 150 °C increased the equivalent Ag_2_O conversion based on CO_2_ to 33.3%, while the equivalent conversion calculated from H_2_O production reached 92.4%. This substantial H_2_O generation at 150 °C suggests that catalytic dehydration is the dominant pathway, effectively utilizing the alcohol’s oxygen atoms to form H_2_O.

The volatile organic compounds (VOCs) generated during the reaction of Ag_2_O with the model alcohols (1:5 mol/mol) were analyzed using headspace GC-MS at 150 °C for 10 min to detect reaction intermediates. The resulting mass spectra (Figure 14) revealed complex fragmentation patterns, the details of which are summarized in Table 3. The chromatogram for 1-decanol showed the main reactant peak at 13.78 min (*m*/*z* 158; fragments: 140, 112, 97, 83, 70, 55, 40) (Figure 14.A). The primary oxidation product, decanal, appeared at 10.64 min (*m*/*z* 156; fragments: 138, 128, 112, 95, 82, 70, 57). Next, formation of several hydrocarbons was observed. A major peak occurred after 3.00 min (*m*/*z* 126; fragments: 111, 97, 83, 69, 56, 43) and was identified as 1-nonene. The peak immediately following at 2.55 min (*m*/*z* 128; fragments: 99, 85, 71, 57, 43) corresponds to nonane. The earliest peaks at 1.11 and 0.79 min were attributed mainly to CO_2_ and small hydrocarbons. Smaller peaks appearing between the nonane and CO_2_ regions were identified as 1-octene (2.06 min), octane (1.78 min), and 1-heptene (1.52 min). This elution pattern mirrors the nonene/nonane sequence, which is indeed a very plausible degradation pathway.

**Figure 14:**
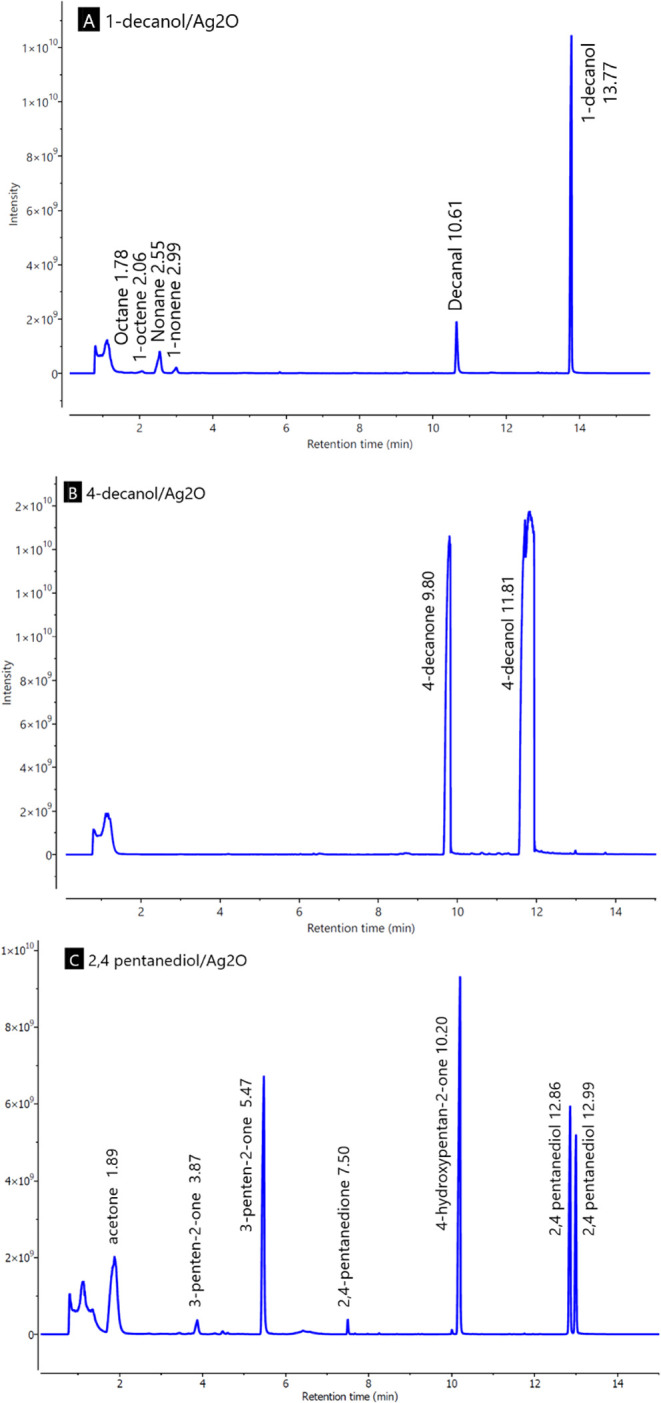
(A) GC-MS headspace analysis of 1-decanol/Ag_2_O (1:5 mol/mol) at 150 °C for 10 min. (B) GC-MS headspace analysis of 1-decanol/Ag_2_O (1:5 mol/mol) at 150 °C for 10 min. (C) GC-MS headspace analysis of 1-decanol/Ag_2_O (1:5 mol/mol) at 150 °C for 10 min.

**Table 3:** GC-MS Main Compounds Found with Their Respective Retention Times, Molecular Ions, and Major Fragments

Reactant	Compound Identified	Retention Time (min)	Molecular Ion (*m*/*z*)
1-decanol	1-decanol (reactant)	13.77	158
	decanal	10.61	156
	1-nonene	2.99	126
	nonane	2.55	128
	1-octene	2.06	112
	octane	1.78	114
4-decanol	4-decanol (reactant)	11.81	158
	4-decanone	9.80	156
2,4-pentanediol	2,4-pentanediol (isomers)	12.86/12.99	104
	4-hydroxypentan-2-one	10.20	102
	2,4-pentanedione	7.50	100
	3-penten-2-one (isomers)	3.87/5.47	84
	acetone	1.89	58

The reaction of 4-decanol showed the parent peak at 11.81 min (*m*/*z* 158; fragments: 157, 140, 115, 96, 83, 69, 54, 43) (Figure 14.B). This was followed by a single major product, 4-decanone, at 9.80 min (*m*/*z* 156; fragments: 112, 99, 86, 70, 58, 42). No other major peaks were found, with the final peaks attributed essentially to CO_2_.

The 2,4-pentanediol reactant appeared as two distinct isomeric peaks after 12.99 min (*m*/*z* 104; fragments: 89, 71, 45) and after 12.86 min (*m*/*z* 104; fragments: 89, 71, 42), corresponding to racemic and meso forms (Figure 14.C). Several oxidation and dehydration products were identified: 4-hydroxypentan-2-one appeared at 10.20 min (*m*/*z* 102; fragments: 84, 69, 58, 45), followed by 2,4-pentanedione at 7.50 min (*m*/*z* 100; fragments: 99, 85, 57, 43). Moreover, dehydration yielded two isomers of 3-penten-2-one: the first at 5.47 min (*m*/*z* 84; fragments: 68, 53, 41) and the second at 3.87 min (*m*/*z* 84; fragments: 69, 53, 41). Finally, the large acetone peak appeared after 1.89 min (*m*/*z* 58; fragment: 43) which indicated chain cleavage.

The qualitative profile of the volatile byproducts (GC-MS) correlates strongly with the quantitative oxygen mass balance determined by GC-TCD and KFT. In the case of 1-decanol, the detection of a homologous series of hydrocarbons is consistent with the formation of a reactive aldehyde intermediate and subsequent deep oxidation. Unlike ketones, aldehydes are susceptible to oxidative decarboxylation and chain scission. Conversely, the absence of such hydrocarbons in the 4-decanol reaction highlights the stability of the ketone intermediate; however, the significant quantity of CO_2_ observed suggests that complete oxidation of the ketone chain still occurred at both low and high temperatures. For monoalcohols, the combined oxygen content in the generated CO_2_ and H_2_O accounts accurately for approximately 100% of the oxygen available in the initial Ag_2_O. This stoichiometric agreement indicates that for 1-decanol and 4-decanol, H_2_O is produced primarily through oxidative reactions rather than a dehydration reaction.

In the case of 2,4-pentanediol, we observed products of both single and double oxidation (hydroxy-ketone and 2,4-pentanedione). This is likely linked to sequential oxidation steps, as indicated above in the thermal and gas evolution studies. Moreover, the formed 2,4-pentanedione is documented for its capability to chemisorb onto metal particles and to form complexes,^52–54^ potentially influencing the morphology of the solid transforming Ag particles. Crucially, the detection of 3-penten-2-one is a definitive indication for the presence of a dehydration reaction. This specific pathway accounts for the excess H_2_O produced in this system. The formation of the resulting unsaturated ketone involves the elimination of a hydroxyl group from the organic substrate, confirming that the diol system converts two sources of oxygen, namely from Ag_2_O and alcohol.

The substantial generation of CO_2_ across all model systems points to the diradical nature of the oxygen species released from the Ag_2_O. Atomic oxygen (O) is a highly oxidative species that can drive the formation of full oxidation products. This aligns with our observations for alkanes^12^ and those reported in another study.^51,55^

### 4.5 Ag (Ion) Mobility in Model Liquids

While the reduction of Ag_2_O by alkanes is proposed to be a solid-state reaction,^12^ previous studies have suggested that in alcohol media metal salts partially dissolve to form a mobile phase that facilitates the reaction,^14,56^ and specially also in aqueous media. Understanding this mobility is critical for the extrusion process, as the dissolution and subsequent nucleation of Ag species determine whether nanoparticles can form, whereas purely solid-state reactions would strictly mandate the use of nanoscale precursors. To determine the existence of solvated Ag species (such as Ag^+^) prior to reaction initiation, the solubility of Ag_2_O in H_2_O and dry model liquids was quantified using ICP-OES (Table 4).

**Table 4:** Measured Ag^+^ Concentrations (mg/L) Following the Addition of Ag_2_O to H_2_O, Anhydrous Alcohols, and Aqueous 2,4-Pentanediol at Room Temperature

Solvent	Concentration mg/L
H_2_O	25.5
1-decanol	0.16
4-decanol	0.17
2,4 pentanediol	0.11
2,4 pentanediol/H_2_O 1:6 (mol/mol)	5.03

Consistent with literature values, the solubility of Ag_2_O in pure H_2_O at room temperature was found to be 25.8 mg/L, see reference.^57^ While measurable, this value is orders of magnitude lower than that of common Ag precursors used in nanoparticle synthesis, such as AgNO_3_ (solubility 2.3 × 10^6^ mg/L), see reference.^58^ In contrast, the solubility of Ag_2_O in the dry model alcohols was negligible. Analysis revealed Ag concentrations of only 0.16 mg/L in 1-decanol, 0.17 mg/L in 4-decanol, and 0.11 mg/L in 2,4-pentanediol. These values indicate that Ag_2_O is nearly insoluble in the dry alcohols, suggesting that the redox reaction must be heterogeneous rather than homogeneous. However, the addition of H_2_O significantly alters this solubility profile. When H_2_O was added to 2,4-pentanediol (1:6 mol/mol), the dissolved Ag concentration increased to 5.03 mg/L. This suggests that while the reaction initiates at the solid–liquid interface, the in situ generation of H_2_O during the reduction process (as confirmed by KFT) may eventually create solvated Ag.

To further test the mobility of Ag species, a diffusion experiment was conducted using a setup with two open vials submerged side-by-side in a sealed reactor, filled with dry 2,4-pentanediol. One vial contained solid Ag_2_O and the other solid NaCl. In case of the presence of significant amounts of mobile Ag^+^ ions, these ions would diffuse through the liquid medium and readily react with Cl^–^, precipitating as silver chloride (AgCl) in the second vial. After 1 week in the dry and sealed reactor, EDX analysis of the NaCl vial revealed no traces of AgCl (Figure 15). This absence of precipitate confirms that Ag ions have very limited mobility in dry alcohol media, reinforcing the conclusion that the initiation Ag_2_O of reduction is, under dry conditions, primarily a heterogeneous surface reaction rather than a homogeneous solution process.

**Figure 15:**
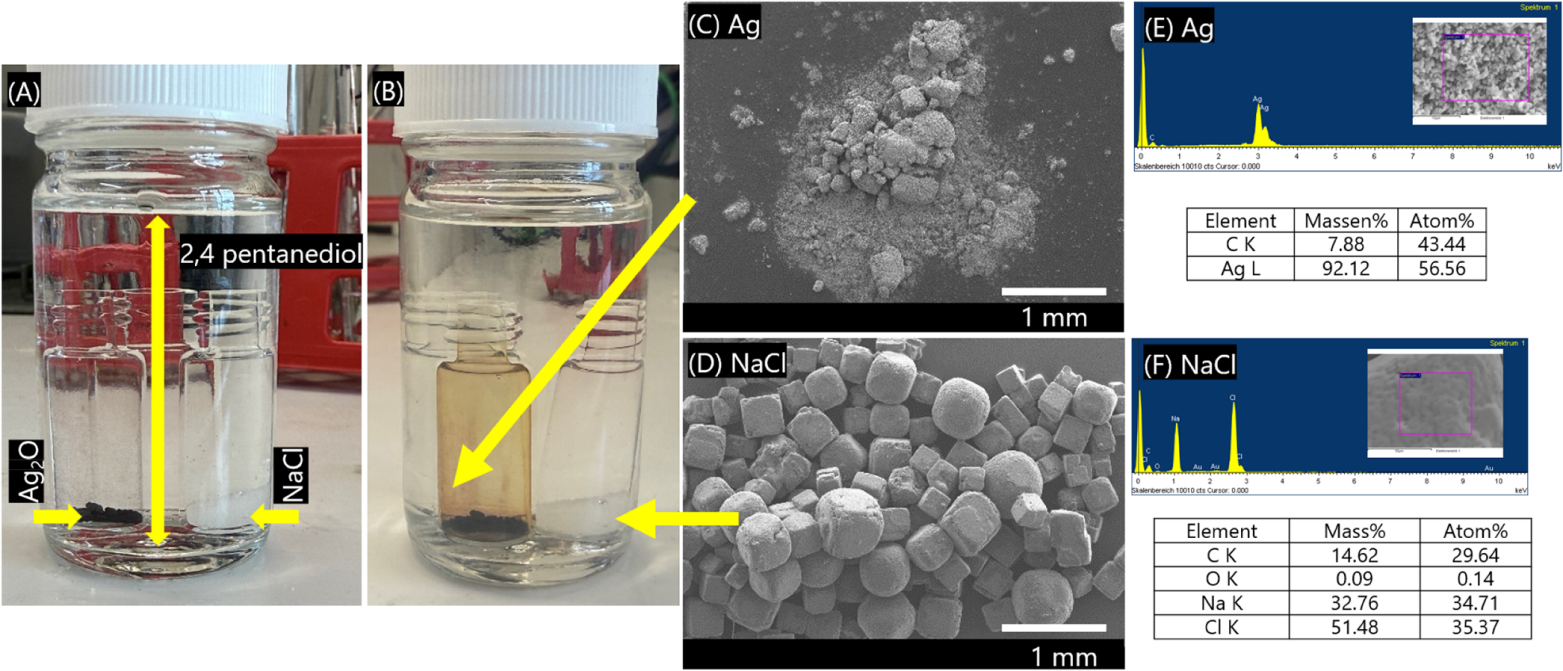
Diffusion experiment in 2,4-pentanediol: (A) Initial experimental setup of 2,4-pentanediol with submerged vials containing Ag_2_O (right) and NaCl (left). (B) Visual observation of the container after 1 week. (C, E) SEM micrograph and EDX spectrum, respectively, of the residual solids from the Ag_2_O vial. (D, F) SEM micrograph and EDX spectrum of the residual solids from the NaCl vial.

SEM analysis of the diffusion experiment confirmed localized growth on the glass walls and a complete absence of the Ag film at the rim top (Figure 16.A). Macroscopically, a distinct Ag film formed on the interior glass walls of the reaction vial; this deposition was strictly confined to the vertical surfaces and abruptly terminated at the container rim (Figures 16.B and 16.C). As established in the previous section, the initial reaction produces H_2_O. We hypothesize that the in situ H_2_O generation facilitates the mobilization of Ag species (solvated ions) and enables subsequent heterogeneous nucleation along the hydrophilic vertical glass walls.

**Figure 16:**
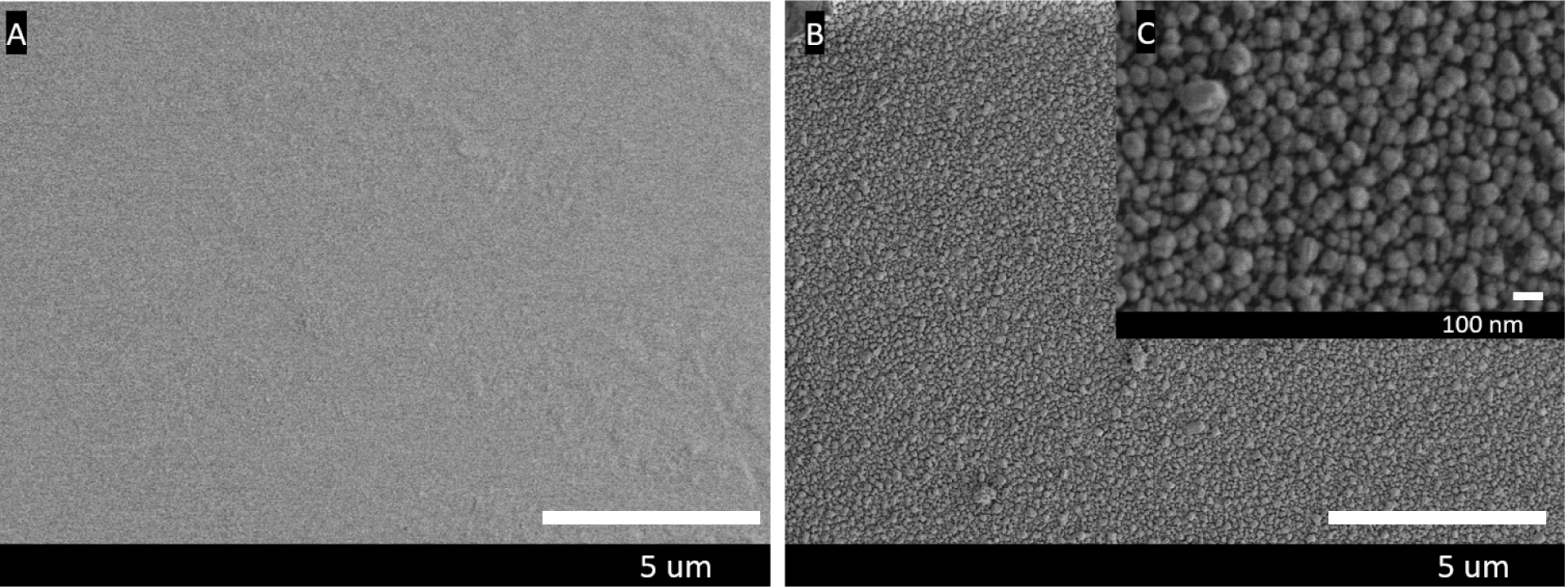
(A) SEM micrographs of the inner vial rim top. (B, C) showing the formed Ag on the glass vertical wall at two magnifications. The Ag particles are mostly spherical in size (diameter 20–200 nm).

The formation of the Ag film on the vial glass wall is described by classical nucleation theory, where the activation energy barrier is lowered by the presence of a substrate, in our case the partially silvered glass wall. The relationship between the Gibbs free energy barrier for heterogeneous nucleation ΔG^het^ and the homogeneous nucleation barrier energy ΔG^hom^, described by the relationship:

$$\Delta G^{het}=f\left(\theta \right)\Delta G^{hom}$$

Where f­(θ) is a geometric shape factor ranging from 0 to 1, determined by the contact angle θ:^59,60^

$$f\left(\theta \right)=\frac{2-3\cos\left(\theta \right)+\cos^{3}\left(\theta \right)}{4}$$

On the vertical glass walls, the depositing Ag phase exhibits high wettability and thus low contact angle, resulting in a negligible shape factor (f­(θ) ≪ 1). This significantly lowers the energy barrier relative to the bulk solution, favoring continuous heterogeneous nucleation and growth of the Ag film on the surface rather than in the bulk liquid. However, the film propagation is observed to stop at the container rim. At this sharp edge, the Ag film encounters a high curvature with angles close to 90° at which the system stops the growth rather than overcoming the elevated energy cost required to advance further.

### 4.6 Morphology of Ag_2_O Reduced in Model Liquids

To further study the nucleation and growth mechanism of the metallic phase, the morphology of the Ag_2_O particles was examined before and after reaction with the model liquids. The pristine Ag_2_O starting material consisted of microsized aggregates (2–5 μm) characterized by irregular spherical geometries and smooth surface textures (Figure 17). To capture the initial stages of Ag formation, the reaction was interrupted after 30 min at 100 °C for all three solvent systems.

**Figure 17:**
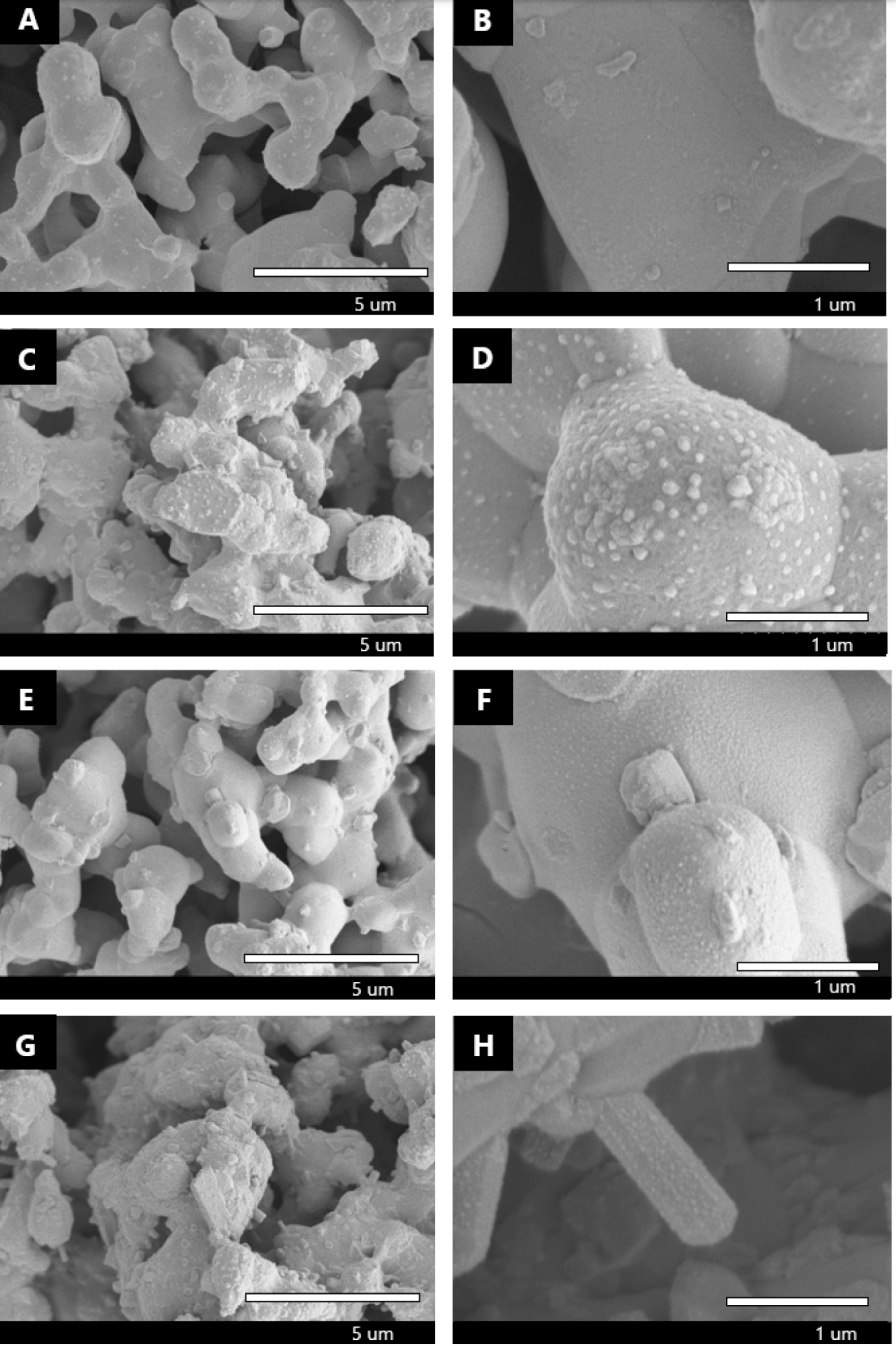
SEM micrographs of Ag_2_O recovered after redox reactions at 100 °C for 30 min. (A, B) Pristine Ag_2_O at low and high magnification, respectively. (C, D) Ag_2_O recovered from the reaction in 1-decanol. (E, F) Ag_2_O recovered from the reaction in 4-decanol. (G, H) Ag_2_O recovered from the reaction in 2,4-pentanediol. Panels (D), (F), and (H) are high-magnification views of the preceding images.

In the case of 1-decanol, distinct spherical nanoparticles were observed nucleating directly on the surface of the parent oxide microparticles. A similar morphological evolution was observed for 4-decanol; however, a clear size difference was noted. The Ag spheres formed in 1-decanol were notably larger than those in 4-decanol, which correlates directly with the kinetics established in the GC-TCD study. Specifically, because 4-decanol reacts slower, the 30 min reaction time captures an early stage of Ag reduction and nucleation. In contrast, due to the faster reaction of 1-decanol the 30 min period includes a more advanced stage of conversion, allowing more time for nanoparticle growth. The rapid oxidation of the primary alcohol drives a higher electron flux to the surface, accelerating the growth rate of the Ag nuclei compared to the slower-reacting secondary alcohol.

The surface morphology in the 2,4-pentanediol system differed from the monoalcohols. While spherical particles were widely distributed, a notable feature was the emergence of high-aspect ratio cylindrical structures protruding from the oxide surface (Figure 17). This anisotropic growth mode is possibly associated with the chelating potential of the diol or its oxidation products (e.g., 2,4-pentanedione) acting as structure-directing agents. Extensive literature on shape-controlled Ag synthesis, notably by the Xia group,^61–63^ has demonstrated that capping agents drive anisotropic growth by selectively passivating distinct crystal surfaces. For instance, poly­(vinyl pyrrolidone) (PVP) binds strongly to {100} facets to induce the growth of Ag rods,^64^ while citrate stabilizes {111} facets to form octahedrons.^64,65^ Drawing on this established work, we propose that the bidentate species in the diol system similarly adsorb onto specific crystallographic planes of the Ag nuclei, hindering isotropic deposition and forcing growth along a single axis to form the observed cylindrical structures.

After 5 h at 100 °C, the distinct morphological features on the bulk oxide surface largely vanished, as extended reaction times promoted the coalescence of seeds into large, sintered aggregates (Figure 18). However, the supernatant retained the unique signatures of the solvent environment. Particles isolated from the monoalcohol supernatants consisted primarily of large clusters of nanospheres. In contrast, the 2,4-pentanediol supernatant contained large rod-like structures (1 μm in length, Figure 19), confirming the observation in the solid phase of the reaction. While discrete nanoparticles were also detected in various control experiments (Figure S10), the specific optimization of these conditions for nanoparticle synthesis remains outside the scope of this work.

**Figure 18:**
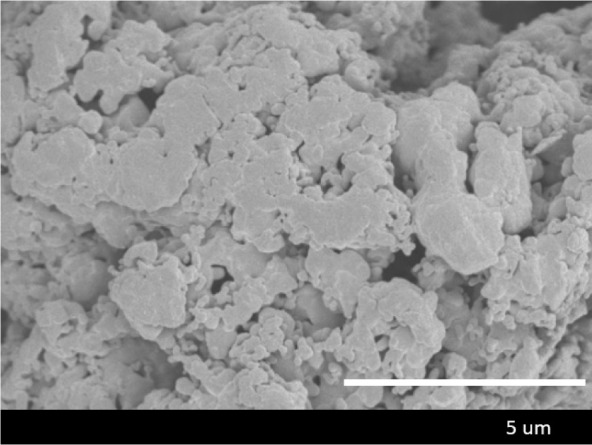
SEM micrographs of sintered Ag particles after prolonged reaction time, 100 °C for 5 h.

**Figure 19:**
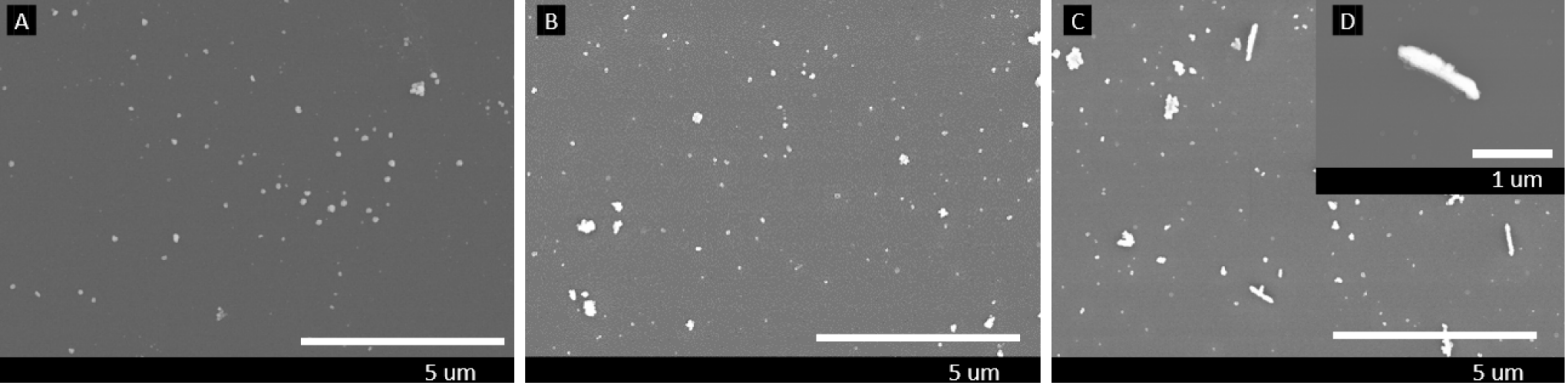
SEM micrographs of Ag particles isolated from the supernatants after the reaction at 100 °C for 5 h. (A) From the reaction of Ag_2_O and 1-decanol. (B) From the reaction of Ag_2_O and 4-decanol. (C) From the reaction of Ag_2_O and 2,4-pentanediol. (D) High-magnification views of (C).

### 4.7 Proposed Reaction Mechanisms

The model liquid systems provided clear insights into the fundamental reactions that govern the polymer extrusion process. Since measuring gases directly during extrusion is difficult, these liquid analogs were essential for identifying H_2_O and CO_2_ as the main byproducts, which directly explains the void formation observed in the extruded composites. The models revealed that the reduction process is exothermic and highly temperature-dependent, with the specific structure of the alcohol dictating the exact reaction pathways. Furthermore, the liquids demonstrated that the reaction is limited by system mobility rather than chemical reactivity. Because the Ag_2_O largely remains in a solid state, this lack of mobility is the main cause of nanoparticle agglomeration in the extruder. Additionally, it was established that dehydration reactions produce intermediate species that help shape and cap the particles, while the water generated during the reaction can potentially enhance silver mobility. Ultimately, by isolating the chemistry from the highly viscous polymer melt, the model systems successfully explain the gas-induced voiding, agglomeration, and particle formation seen during macroscopic extrusion.

In general, the reactions of Ag_2_O with the alcohol model liquids yield either H_2_O-rich or CO_2_-rich product mixtures, a distribution heavily determined by the reaction temperature.

At low temperatures, the dominant pathway is likely an oxidative dehydrogenation reaction. In this process, the alcohol loses hydrogen atoms to the Ag_2_O, generating H_2_O and an aldehyde or ketone. It is worth noting that Ag has been documented to catalyze dehydrogenation reactions.^66,67^ In our specific system, this dehydrogenation mechanism is responsible for the high proportion of H_2_O relative to CO_2_ observed at lower temperatures.

Conversely, higher reaction temperatures and rapid rates consistently favor a radical complete oxidation pathway, leading to C–C bond breakage and increased CO_2_ generation. This combustion-like (complete oxidation) behavior could be linked to atomic oxygen^55^ (O) released from Ag_2_O. Assuming complete oxidation with sufficient oxygen, an alcohol such as 1-decanol then reacts according to the following equation:

$$C_{10}H_{21}OH+30\left[O\right]\to 10CO_{2}+11H_{2}O$$

This stoichiometric distribution results in totally 31 oxygen atoms in the products: 20 allocated to CO_2_ and 11 to H_2_O. Consequently, approximately 65% of the product oxygen is attributed to CO_2_ and 35% to H_2_O. A similar oxygen distribution has been published previously for 1-tetradecanol,^51^ where the reactions occurred at elevated temperatures and rapid heating rates, conditions that favor a complete oxidation reaction.

Therefore, the low temperature dehydrogenation mechanism proposed below will always compete with this radical driven complete oxidation pathway depending on the thermal conditions applied. Below is the detailed process for each specific alcohol evaluated in this study at high temperature, which is applicable to extrusion conditions, see Scheme 1.

**Scheme 1:**
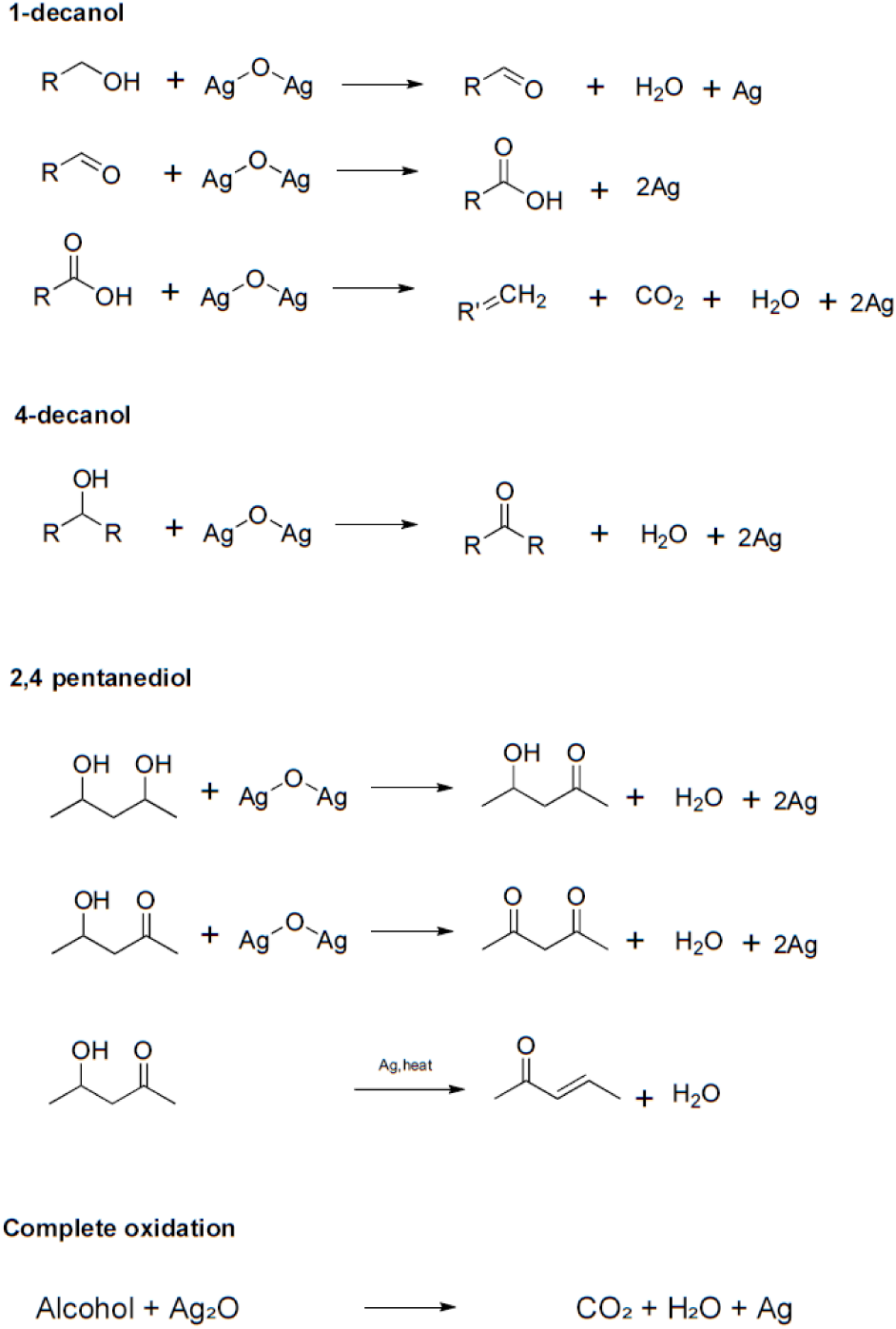
Schematic of Alcohol Oxidation by Ag_2_O

#### 4.7.1 Redox Reaction of 1-Decanol with Ag_2_O

The reaction of 1-decanol with Ag_2_O is characterized by a rapid autocatalytic reaction, with aldehyde formation and the detection of chain shortened hydrocarbons, and a precise 1:1 stoichiometric ratio between the oxygen atoms found in CO_2_ and H_2_O. We propose two main paths following the main step mechanism:

##### 4.7.1.1 Oxidative Dehydrogenation

The reaction initiates with the abstraction of α-hydrogen atoms by Ag_2_O surface oxygen atoms, converting 1-decanol to decanal with the generation of H_2_O and Ag.

$$R-CH_{2}OH+Ag_{2}O\to R-CHO+H_{2}O+2Ag$$

##### 4.7.1.2 Oxidation to Carboxylic Acid

The reactive aldehyde intermediate undergoes rapid oxidation on the Ag_2_O surface to forming carbonic acids and Ag.

$$R-CHO+Ag_{2}O\to R-COOH+2Ag$$

##### 4.7.1.3 Oxidative Decarboxylation

At high reaction temperatures, the acids can undergo decarboxylation. At high reaction temperatures, the acids can undergo oxidative decarboxylation. Although written as a single step, this net reaction likely proceeds sequentially, where decarboxylation precedes alkene formation.

$$R-CH_{2}-CH_{2}-COOH+Ag_{2}O\to R-CH=CH_{2}+CO_{2}+H_{2}O+2Ag$$

Then the total reaction of steps a, b, and c would be

$$R-CH_{2}-CH_{2}-CH_{2}OH+3Ag_{2}O\to R-CH=CH_{2}+CO_{2}+2H_{2}O+6Ag$$

This mechanism reveals an equal distribution of oxygen atoms between CO_2_ and H_2_O, which matches the experimental values obtained from the TCD and KFT analysis at 150 °C.

##### 4.7.1.4 Complete Oxidation

A complete oxidation reaction could occur with any of the formed intermediates, generating a higher ratio of oxygen atoms in CO_2_ than in H_2_O which would be the case at high temperature as reported by others.^51^

$$C_{10}H_{21}OH+30Ag_{2}O\to 10CO_{2}+11H_{2}O+60Ag$$

The combination of a, b, and c step accounts for both the dominant aldehyde and alkenes/alkanes presence in the GC-MS and the nearly 50/50 oxygen distribution observed in the TCD and KFT analyses. However, the complete oxidation (step d) can result in a theoretical 65/35 oxygen distribution which would be the case at higher temperatures.

#### 4.7.2 Redox Reaction of 4-Decanol with Ag_2_O

Although the reaction main products are similar to 1-decanol, no alkenes/alkanes were observed. We propose below paths:

##### 4.7.2.1 Oxidative Dehydrogenation

The secondary alcohol undergoes oxidative dehydrogenation to form 4-decanone. This process is the main path for the H_2_O being generated.

$$R-CH\left(-OH\right)-R_{2}+Ag_{2}O\to R-CH\left(\mathrm{=O}\right)-R_{2}+H_{2}O+2Ag$$

##### 4.7.2.2 Complete Oxidation

In the case of 4-decanol, the initial oxidative dehydrogenation yields a ketone. Since ketones lack the reactive aldehydic proton, they are significantly less susceptible to further mild oxidation compared to aldehydes. Consequently, the CO_2_/ H_2_O product ratio remains constant across the tested temperature range. Further degradation of this stable ketone intermediate requires C–C bond cleavage, which occurs primarily through the complete oxidation mechanism.

#### 4.7.3 Redox Reaction of 2,4-Pentanediol with Ag_2_O

The reaction of 2,4-pentanediol, produced large amount of H_2_O and confirmed that complete oxidation is not a main path. Thus, two reaction paths are proposed:

##### 4.7.3.1 Oxidative Dehydrogenation

The reaction of 2,4-pentanediol with Ag_2_O generated 4-hydroxypentan-2-one (hydroxy ketone), H_2_O and Ag.

$$CH_{3}CH\left(OH\right)CH_{2}CH\left(OH\right)CH_{3}+Ag_{2}O\to CH_{3}C\left(\mathrm{=O}\right)CH_{2}CH\left(OH\right)CH_{3}+H_{2}O+2Ag$$

##### 4.7.3.2 Secondary Dehydrogenation Oxidation

Next, the hydroxy ketone reacts again with Ag_2_O generating a diketone, H_2_O and Ag.

$$CH_{3}C\left(\mathrm{=O}\right)CH_{2}CH\left(OH\right)CH_{3}+Ag_{2}O\to CH_{3}C\left(\mathrm{=O}\right)CH_{2}C\left(\mathrm{=O}\right)CH_{3}+H_{2}O+2Ag$$

Each mole of the diol contains two hydroxyl groups compared to a monoalcohol which could participate in two oxidative dehydrogenation steps resulting in more H_2_O.

##### 4.7.3.3 Dehydration

Furthermore, GC-MS analysis indicated the presence of 3-penten-2-one, which was generated via a subsequent dehydration reaction of the intermediate hydroxy ketone formed in the first step. Together, these steps explain the high amount of H_2_O evolution. The hydroxy ketone formed in step *a* can undergo underwent catalyzed dehydration due to elevated temperature and Ag as catalyst.

$$CH_{3}C\left(\mathrm{=O}\right)CH_{2}CH\left(OH\right)CH_{3}\overset{Ag,\Delta }{\to }CH_{3}C\left(=O\right)CH=CHCH_{3}+H_{2}O$$

##### 4.7.3.4 Complete Oxidation

Although not the dominant pathway, the CO_2_ observed in the GC-TCD experiments and the various fragmentation products detected via GC-MS indicate C–C bond cleavage, pointing to a complete oxidation mechanism. Any of the intermediate organic products could undergo this subsequent complete oxidation, which is necessary to account for the total generated CO_2_.

$$CH_{3}CH\left(OH\right)CH_{2}CH\left(OH\right)CH_{3}+14Ag_{2}O\to 5CO_{2}+6H_{2}O+28Ag$$

To account for the highly excess of H_2_O, we propose that the newly formed metallic Ag itself thermally catalyzes the dehydrogenation/dehydration processes (step *a*,*b*,*c*) as Ag is well-documented to catalyze the dehydrogenation and dehydration of alcohols.^66,67^ This catalytic enhancement is especially pronounced in the case of 2,4-pentanediol due to its ability to coordinate with and cap Ag nanoparticles more efficient than monoalcohols.

### 4.8 Application Cases of Ag_2_O Reduction in PVA

To demonstrate practical application of this in situ reduction chemistry and address limitations regarding Ag mobility in bulk polymers, Ag-PVA nanocomposite films were synthesized via a water-assisted method (Figure S3). Characterization confirmed the in situ generation of well-dispersed Ag nanoparticles within the polymer matrix, with sizes ranging from 30–50 nm (Figure 20.A and 20.B). Furthermore, the fabricated PVA/Ag film appears yellow (Figure 20.C). UV–vis spectroscopy of the film exhibited a distinct surface plasmon resonance (SPR) peak centered at 401 nm; the narrow width of this absorption curve indicates a narrow particle size distribution.

**Figure 20:**
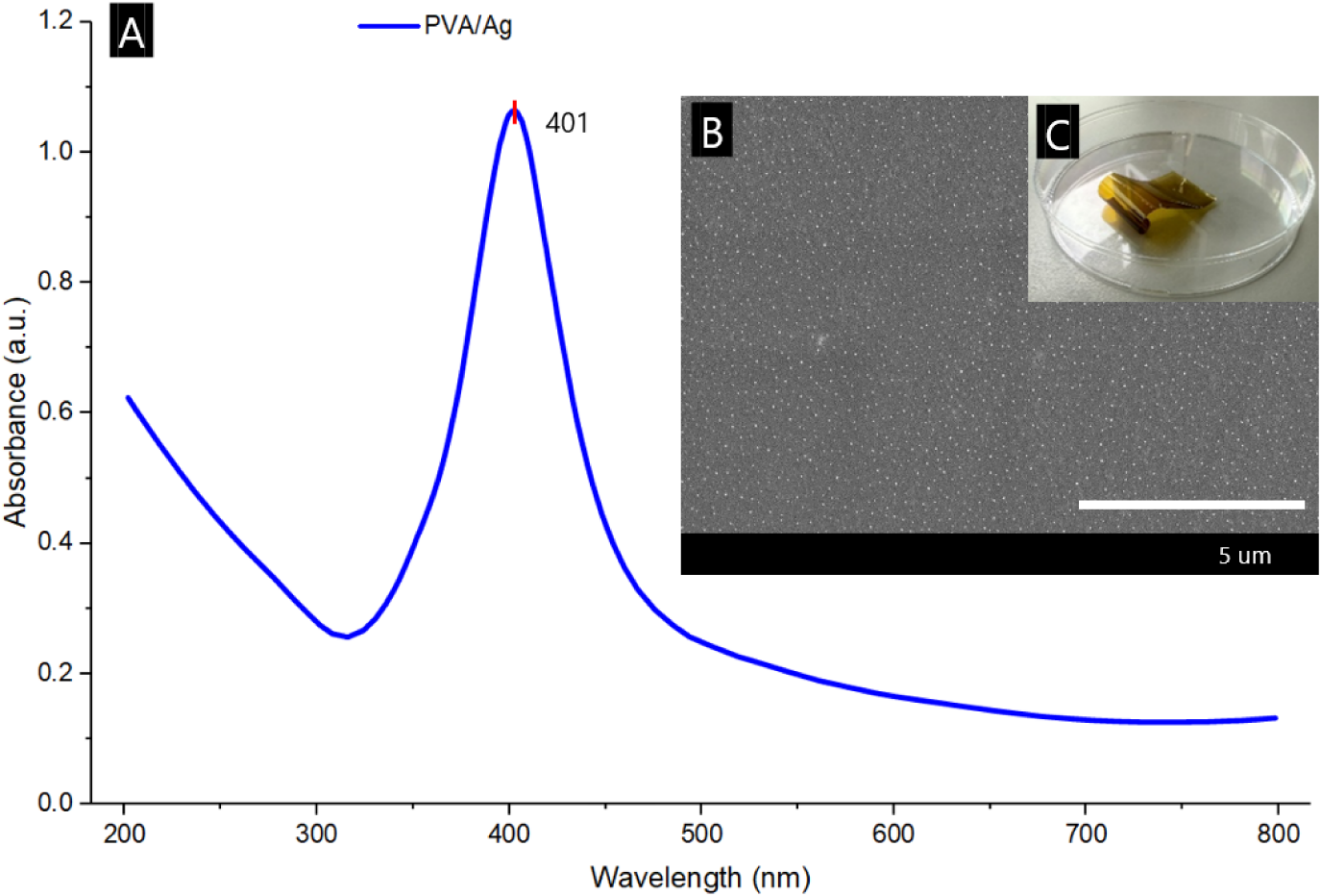
(A) UV–vis absorption spectrum of a film prepared via in situ reduction of Ag_2_O in aqueous PVA at 60 °C, showing the characteristic SPR peak of Ag nanoparticles. (B) SEM micrograph displaying the morphology and distribution of the Ag nanoparticles within the PVA matrix. (C) Image demonstrating the characteristic yellow coloration of the resulting PVA/Ag film.

The Ag+ ions have demonstrated significant effectiveness as both antimicrobial and antiviral agents, primarily due to their multimodal mechanisms of action.^68^ Thus, Ag nanoparticles have been used extensively in polymer film applications.^69,70^ Recent studies have demonstrated that incorporating green-synthesized Ag nanoparticles into blends of PVA and fluorinated polymers significantly enhances their antimicrobial capabilities, making them highly suitable for wound dressing applications.^71–73^ Moreover, Ag^+^ ions exhibit a strong inhibitory effect against various pathogens, including Gram-negative bacteria (*Escherichia coli*), Gram-positive bacteria (*Staphylococcus aureus*), and fungal yeast (*Candida albicans*).

To assess the functional efficacy of the synthesized nanocomposite films, an antibacterial diffusion assay was conducted using a neat PVA film as a control. Antibacterial activity was evaluated against *Escherichia coli* ATCC 8739, a standard Gram-negative bacterial strain for susceptibility test. Despite a low precursor loading of only 0.5% w/w Ag_2_O relative to the PVA matrix, the composite film demonstrated strong antimicrobial activity. The nanocomposite samples yielded a clear inhibition zone, whereas the control PVA samples exhibited no inhibitory effect (Figure 21). This pronounced activity at such low loading levels is attributed to the high surface area to volume ratio of the small, in situ generated Ag nanoparticles, which facilitates a more efficient release of biocidal Ag ions. A supplementary figure of the antimicrobial samples is shown in (Figure S11)

**Figure 21:**
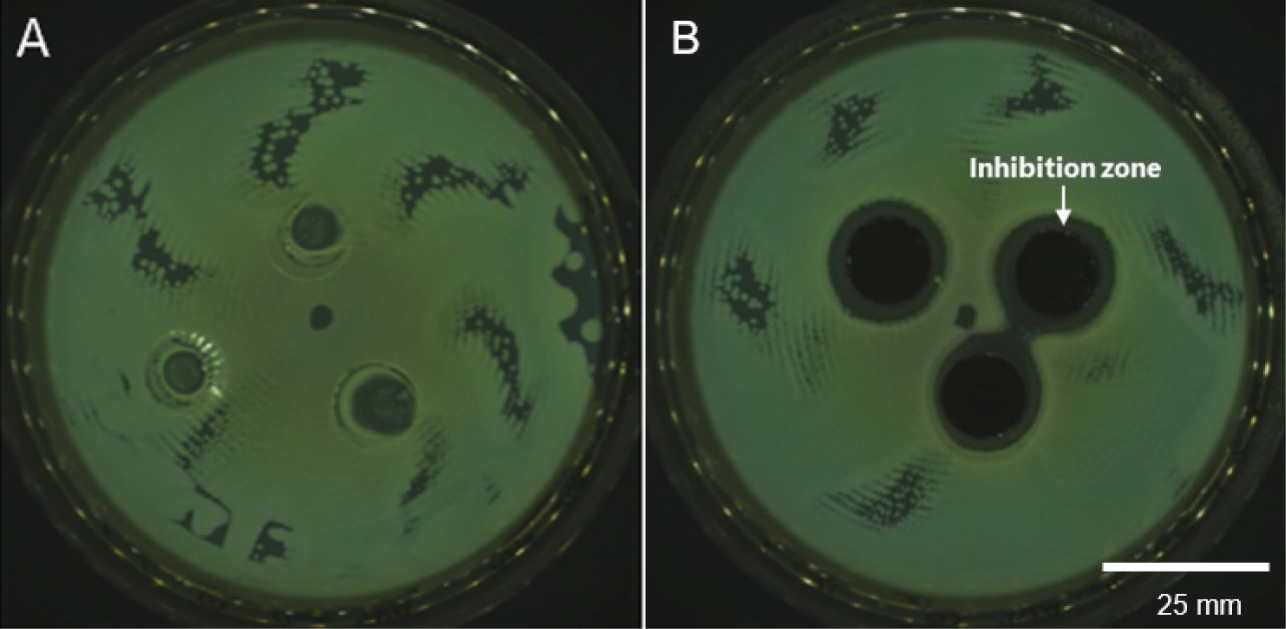
Antimicrobial agar diffusion test against *E. coli* ATCC 8739. (A) A neat PVA control film did not exhibit an inhibition zone, (B) PVA/Ag nanocomposite film (0.5% w/w Ag_2_O) demonstrated a clear zone of inhibition. A digital image of the samples in visible light is shown in Figure S11.

Furthermore, the alignment of nanoparticles to create dichroic optical components was explored.^25^ Composite films were heated to 120 °C and mechanically drawn to 5 times their original length (Figure S4). SEM imaging revealed a distinct unidirectional alignment of the Ag nanoparticles along the drawing axis (Figure 22), in contrast to an unannealed film (Figure 20.B). This alignment induced a dichroic effect (Figure 23), allowing the film to act as a polarizer. Image processing analysis (Figure S5) quantified a darkening effect of approximately 25% when comparing the parallel orientation to the perpendicular orientation between the drawing axis and the polarization direction (Figure 23), suggesting anisotropic alignment of the Ag nanostructures within the PVA matrix.

**Figure 22:**
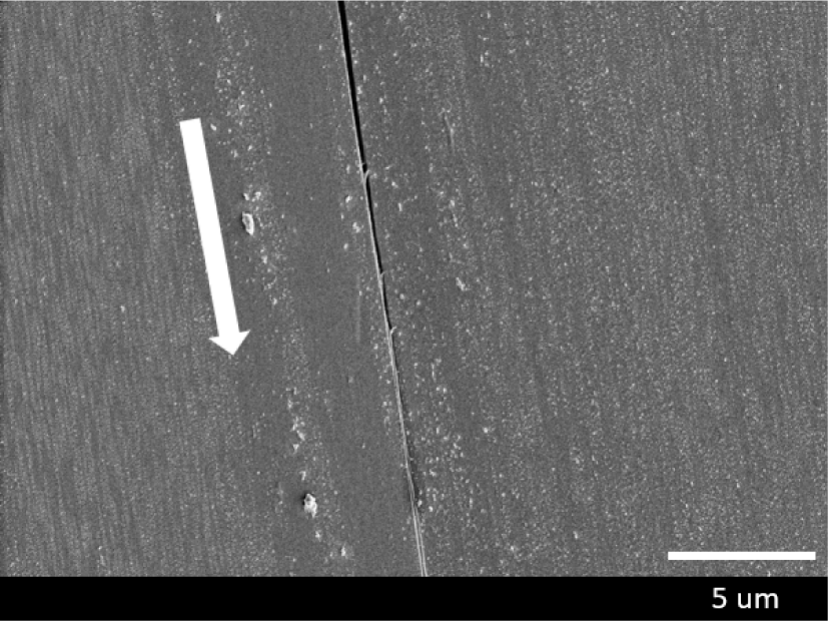
SEM imaging of 5-times drawn PVA/Ag_2_O films, showing unidirectional alignment of Ag nanoparticles along the drawing axis.

**Figure 23:**
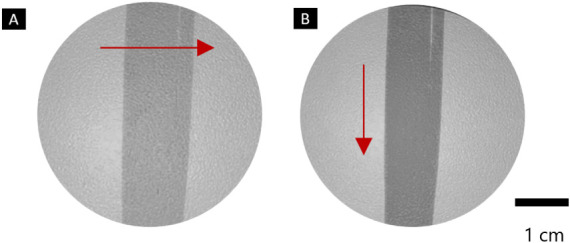
Polarized optical micrographs of a PVA/Ag film drawn to a ratio of 5:1. The images demonstrate the optical anisotropy relative to the draw direction. (A) Micrograph taken with the incident light polarized perpendicular to the draw direction, resulting in higher transmission (bright state). (B) Micrograph taken with the polarization parallel to the draw direction, showing darkening of the film (dark state).

## 5 Conclusions

In conclusion, we have demonstrated that Ag_2_O undergoes a redox reaction during compounding with PVA. Mechanistically, this process is fundamentally limited by mobility. This understanding was achieved through a model system approach with different alcohols, providing critical insights into reaction mechanisms. The primary byproducts of these reactions are H_2_O and CO_2_, with ketones, aldehydes, and alkenes/alkanes appearing as minor products. The ratio between H_2_O and CO_2_ changes based on the alcohol, indicating that the specific alcohol structure plays a major role in the reaction pathways. Two major reactions appear to occur, oxidative dehydrogenation and complete oxidation reactions, the former likely accelerated by the catalytic activity of metallic Ag itself. Notably, a dehydration reaction in the case of 2,4-pentanediol was associated with a capping effect of the 2,4-pentanediol for the nanoparticles. The morphology of Ag reduced in the alcohols is dominated by a surface-solid transformation reaction. The Ag mobility indeed increased slightly with increasing H_2_O levels produced during the reactions. Therefore, as a control strategy, enhancing the system’s mobility either by using a low-melting-temperature PVA or a more soluble precursor salt, could improve nanoparticle dispersion and minimize agglomeration by overcoming the solid–solid diffusion limitations. Regarding applications, this should provide particle morphology control in applications, such as optically polarizing or antimicrobial films. Beyond such applications, this study contributes to a broader fundamental understanding of reactive extrusion using liquid model systems as a methodology for elucidating mechanisms governing nanoparticle generation in polymer melts.

## Supplementary Material



## Notes

Figures showing the particle size distribution (S1 A) and thermal study (S1 B) of Ag_2_O; setups and apparatuses for CO_2_ and H_2_O quantification (S2) and optical analysis of drawn PVA/Ag films (S5); details on dissolved PVA and PVA with Ag_2_O (S3) and the film casting procedure (S4); thermal studies of Ag_2_O (S6) and PVA (S7); reduction in acetic acid (S8); GC-MS analysis of synthesized 4-hydroxypentan-2-one (S9); synthesis of silver nanoparticles in 1-decanol (S10) and a photographic image of antimicrobial agar diffusion test samples (S11) (PDF)
