# Supplementary material for: Silver Oxide Reduction Chemistry in an Alcohol Environment
Source: ACS Omega. 2026 Jun 23;11(26):39293–314. doi: 10.1021/acsomega.6c03865 (PMC13347648; doi:10.1021/acsomega.6c03865)
Supplement: Supplementary file 1 [file ao6c03865_si_001.pdf]

Silver Oxide Reduction Chemistry in an Alcohol Environment

AUTHOR NAMES

Fayez A. Alfayez[a,b] *Simon Ducolombier*[a], *Walter R. Caseri*[b], *Qun Ren*[c], *Sabyasachi Gaan*[a,*], *Manfred Heuberger*[a,b,*]

AUTHOR ADDRESS

[a]Advanced Fibers, Empa Swiss Federal Laboratories for Materials Science and Technology, St Gallen CH-9014, Switzerland

[b]Department of Materials, ETH Zürich, Zürich CH-8093, Switzerland

[c]Laboratory for Biointerfaces, Empa Swiss Federal Laboratories for Materials Science and Technology, St Gallen CH-9014, Switzerland

Sabyasachi Gaan: Sabyasachi.Gaan@empa.ch
Manfred Heuberger: Manfred.Heuberger@empa.ch

KEYWORDS

Silver oxide, 1-decanol, 4-decanol, 2,4 pentanediol, polymer, reduction, mechanism

Table of Contents

1.	Figure S1: Size Distribution of Ag_2O and DSC Thermogram................................	2
2.	Figure S2: GC-TCD and CO2 measurement setup...	3
3.	Figure S3: Photos of PVA solutions..	3
4.	Figure S4: PVA/Ag films..	4
5.	Figure S5: Optical analysis Setup..	5
6.	Figure S6. TGA thermogram of Ag2O	6
7.	Figure S7: DSC thermograms..	7
8.	Figure S8: Silver in DSC Pan..	7
9.	Figure S9: GC-MS chromatogram..	8
10.	Figure S10. Silver nanoparticles wet synthesis Photos......................................	9
11.	Figure S11. Antimicrobial agar diffusion test..	9
12.	References..	10

Supplementary information

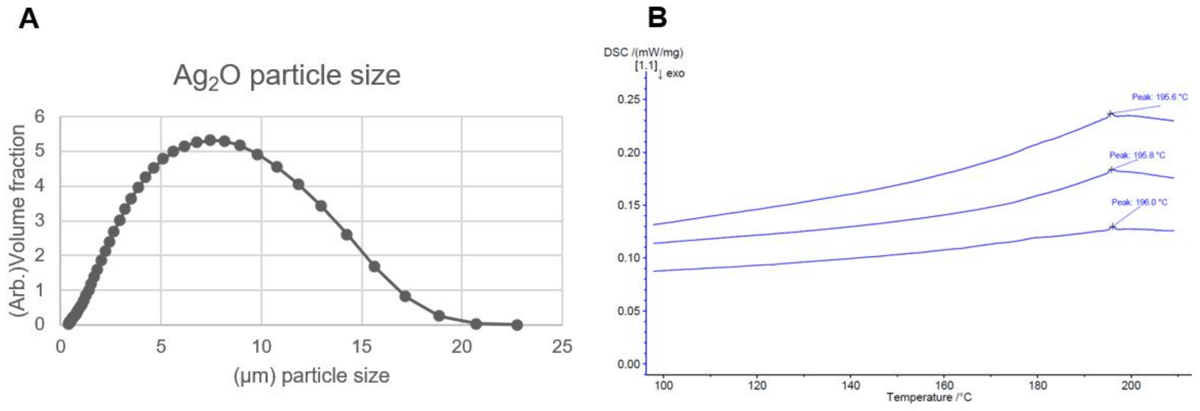

Figure S1. (A) Volume-based particle size distribution curve for Ag_2O used. (B) DSC thermogram of Ag_2O as received without thermal treatment. The conversion of Ag_2CO_3 to Ag_2O occurs at 196 °C at a heating rate of 5 °C/min under N_2 flow.

(A) Ag_2O powder exhibited unimodal size distribution with most particles falling within the 0.5 to 20 micrometer range. The test was conducted using laser diffraction particle size analyzer equipped with a wet dispersion unit. (B)The DSC thermogram of the Ag_2O showed a small but reproducible endothermal peak concluded before 200 °C. The peak is associated with conversion of Ag_2CO_3 to Ag_2O and $CO_{2,}$,[1] thus treatment temperature was selected to be 200 °C.

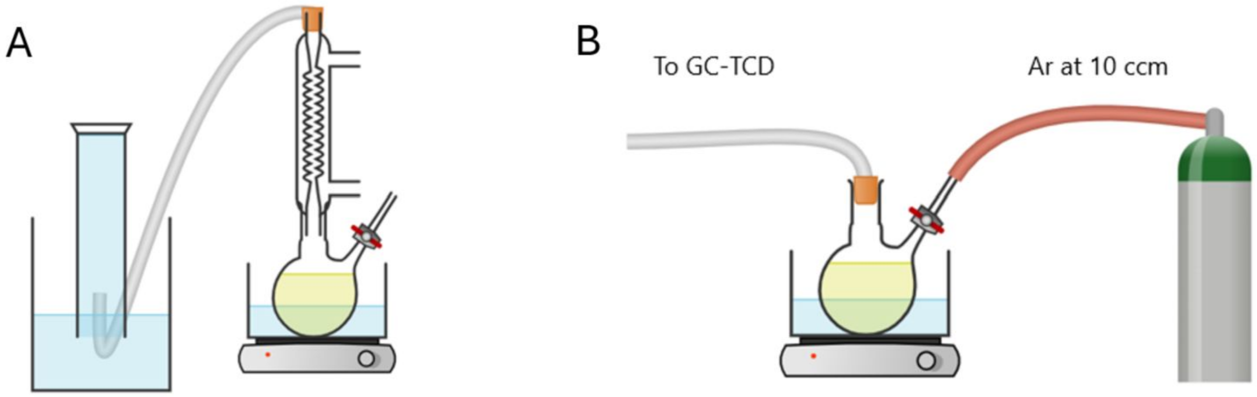

Figure S2. Two configurations used throughout the study. (A) shows the configuration used for measuring CO_2 and H_2O using an inverted graduated cylinder to monitor gas (CO_2) evolution at 150 °C; the remaining liquid in the reaction flask was used to measure H_2O content by KFT. (B) shows the configuration used for CO_2 measurements at 100 °C by GC-TCD.

The two setups used throughout the study for the measurements of CO_2 and H_2O are shown. (A) was used for measurements at 150 °C and (B) was used for measurements at 100 °C.

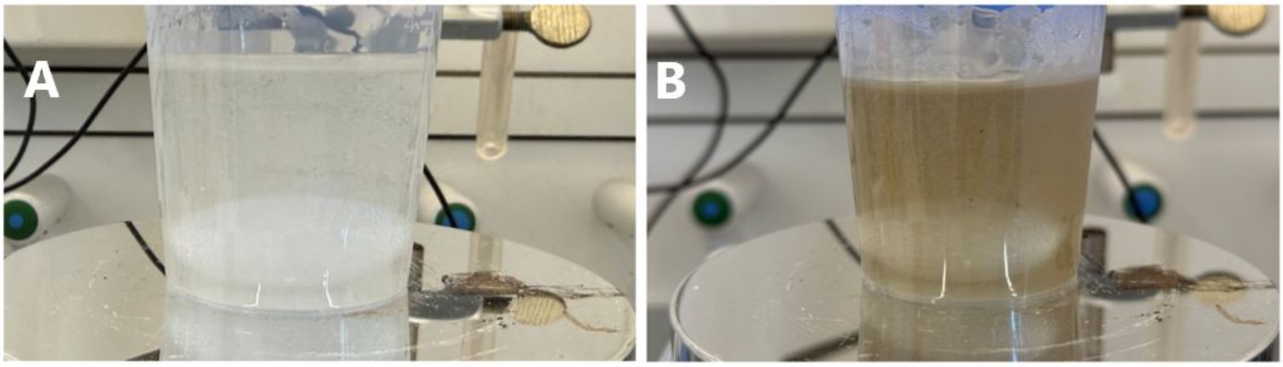

Figure S3. (A) PVA dissolved in H_2O (15% w/v concentration) then Ag_2O was added (0.5% w/w relative to the PVA) at 60 °C. (B) After 5 min silver particles started to form.

The reduction of Ag_2O in dissolved PVA occurred during the mixing step at 60 °C.

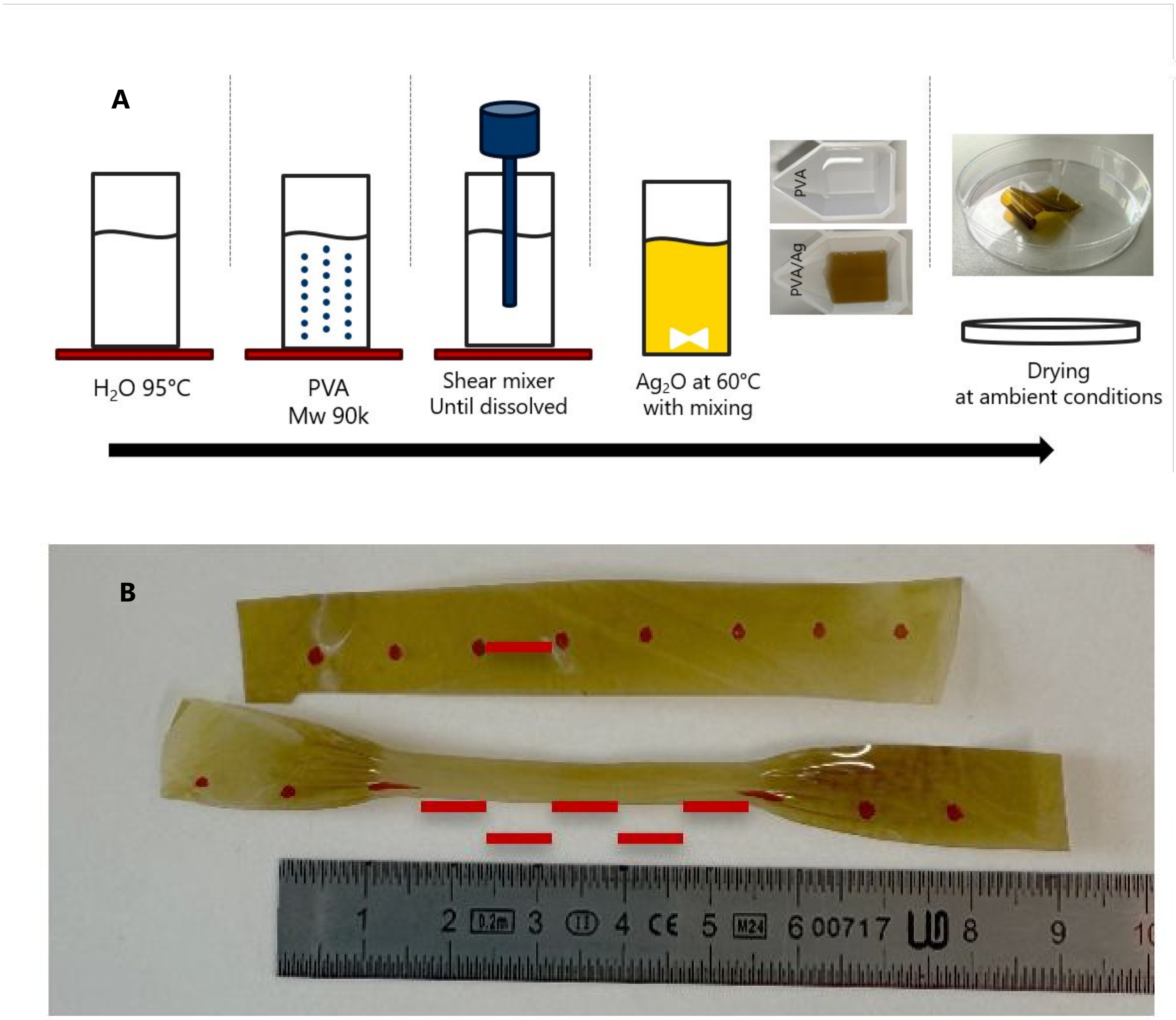

Figure S4. (A) Scheme of the casting procedure of PVA/Ag films before and after drawing. (B) Demonstration of the original and final length of the film

PVA/Ag films were fabricated using a casting procedure (Figure S4.A). The films were heated to 120 °C prior to drawing (Figure S4.B).

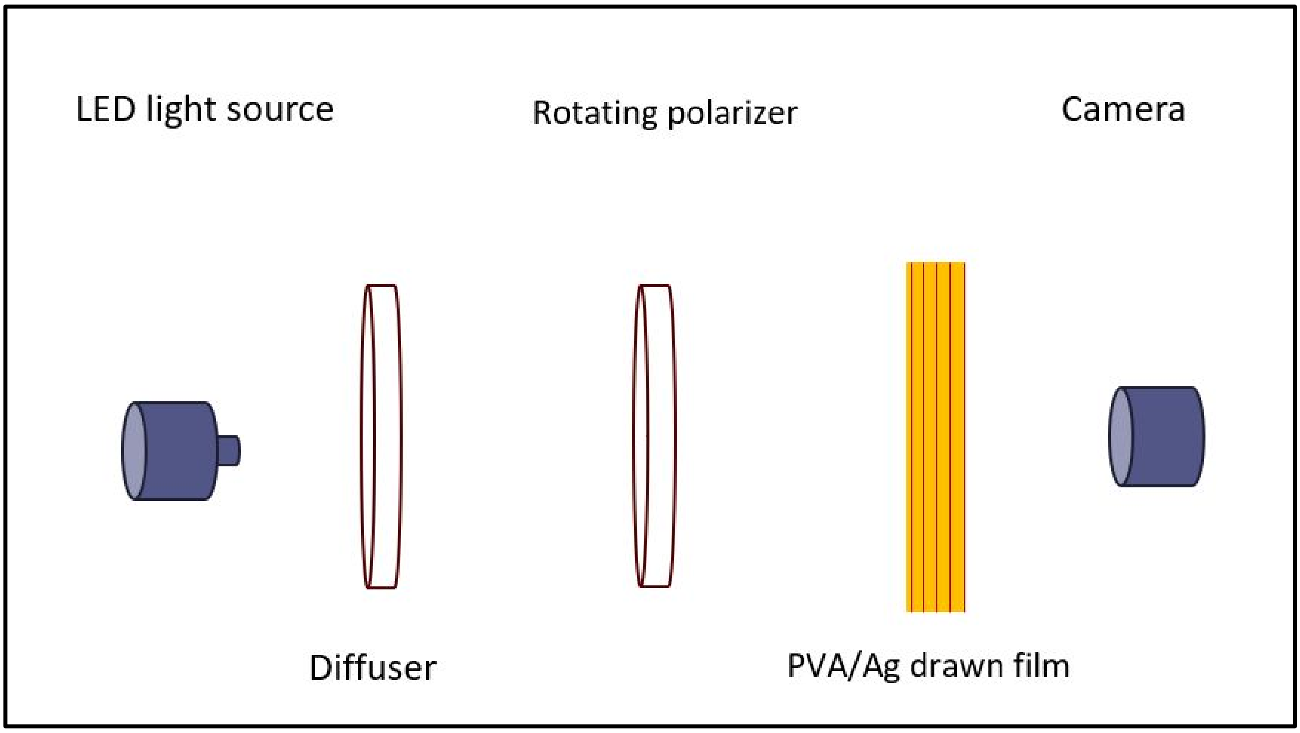

Figure S5. Setup used to analyze the optical effect of drawn PVA/Ag films.

The software Imagej was used to analyze images of two contrasting polarization angles of the incident light with respect to the draw direction of the films (90° and 0°). The obtained data are indicated bleow.

	Mean Intensity
Bright	165.30
Dark image	124.26

The darkening effect was calculated as follows:

$$Darkening\% = \frac{I_{bright} - I_{dark}}{I_{bright}} \times 100$$

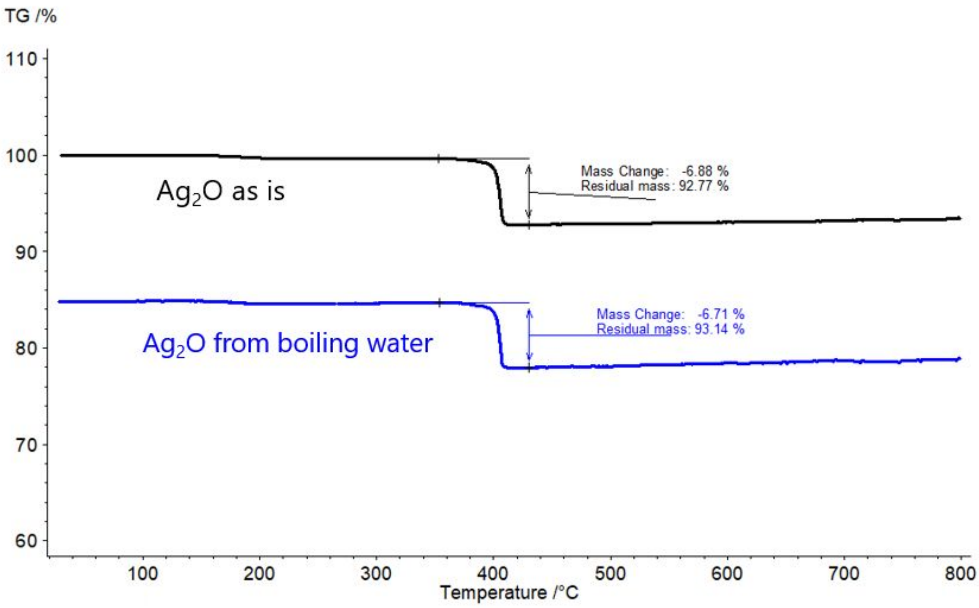

Figure S6. Thermogravimetric analysis (TGA) of Ag_2O at a heating rate of 20 °C/min under N_2 flow. The black curve represents Ag_2O, while the blue curve represents Ag_2O after extraction from boiling water.

To determine if H_2O can reduce the Ag_2O, a quantity of 100 mg Ag_2O was immersed in 100 ml boiling water for 30 min, then extracted and dried. The sample was subsequently analyzed using thermogravimetric tnalysis (TGA) under N_2 atmosphere and compared aginst Ag_2O without treatment. The results showed a mass loss of approximately 6.88% for pure Ag_2O and 6.71% for Ag_2O after treatment. These results closely match the theoretical value of 6.90% for the conversion of Ag_2O to metallic silver. This the experiment demonstrates that H_2O does not reduce Ag_2O.

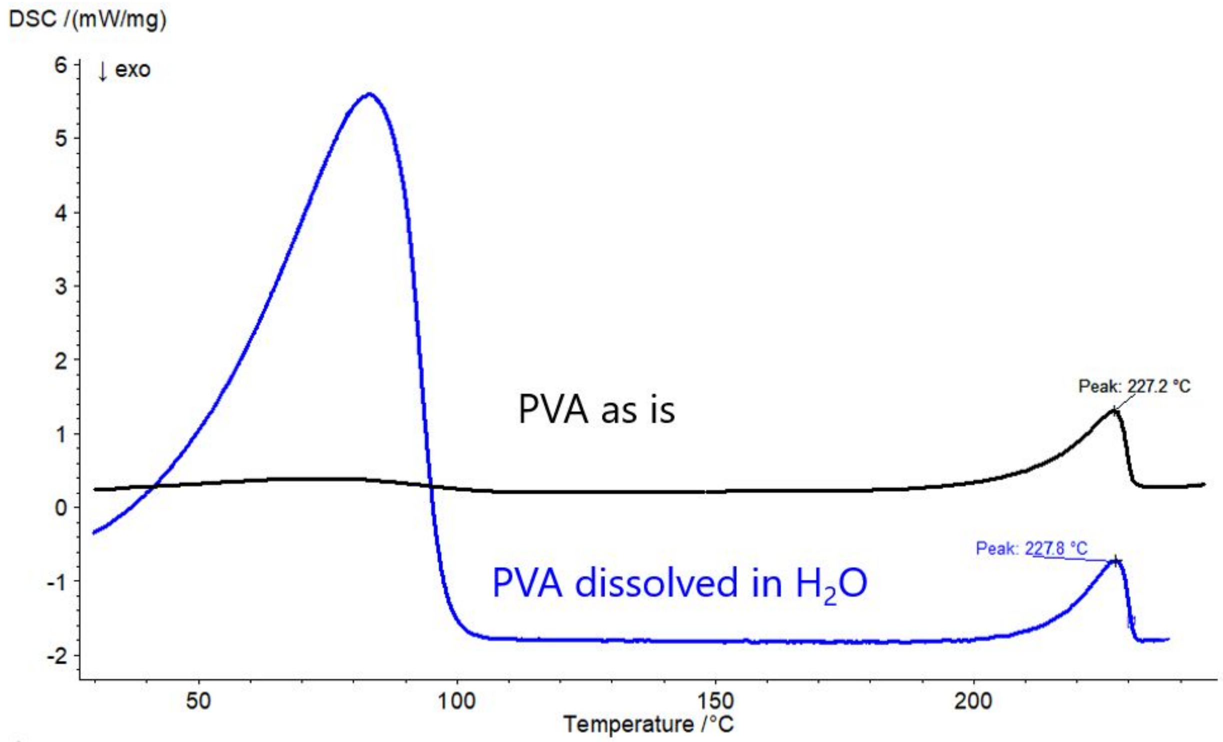

Figure S7. DSC thermogram of pure PVA, represented by the black curve, and PVA dissolved in water (H_2O, 50% w/w concentration), represented by the blue curve. Peak melt temperatures were 227.2 °C and 227.8 °C, respectively, at a heating rate of 5 °C/min under N_2 flow.

The addtion of water to 90k Mw PVA did not change the melting temperature.

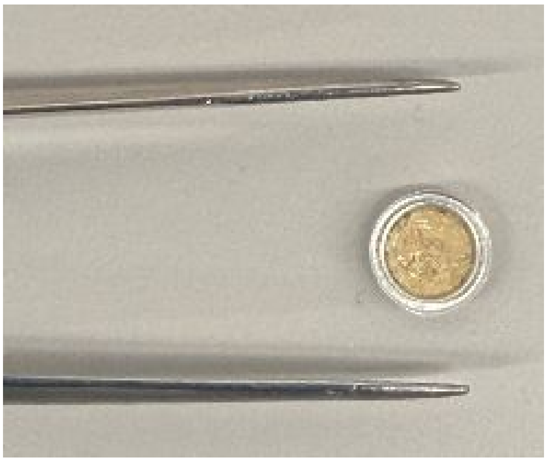

Figure S8. The addition of acetic acid to silver oxide resulted in the immediate conversion of the oxide to elemental silver. This reaction occurred during the preparation of the DSC crucible.

Shows that the acetic acid which immediate redude silver oxide upon contact.

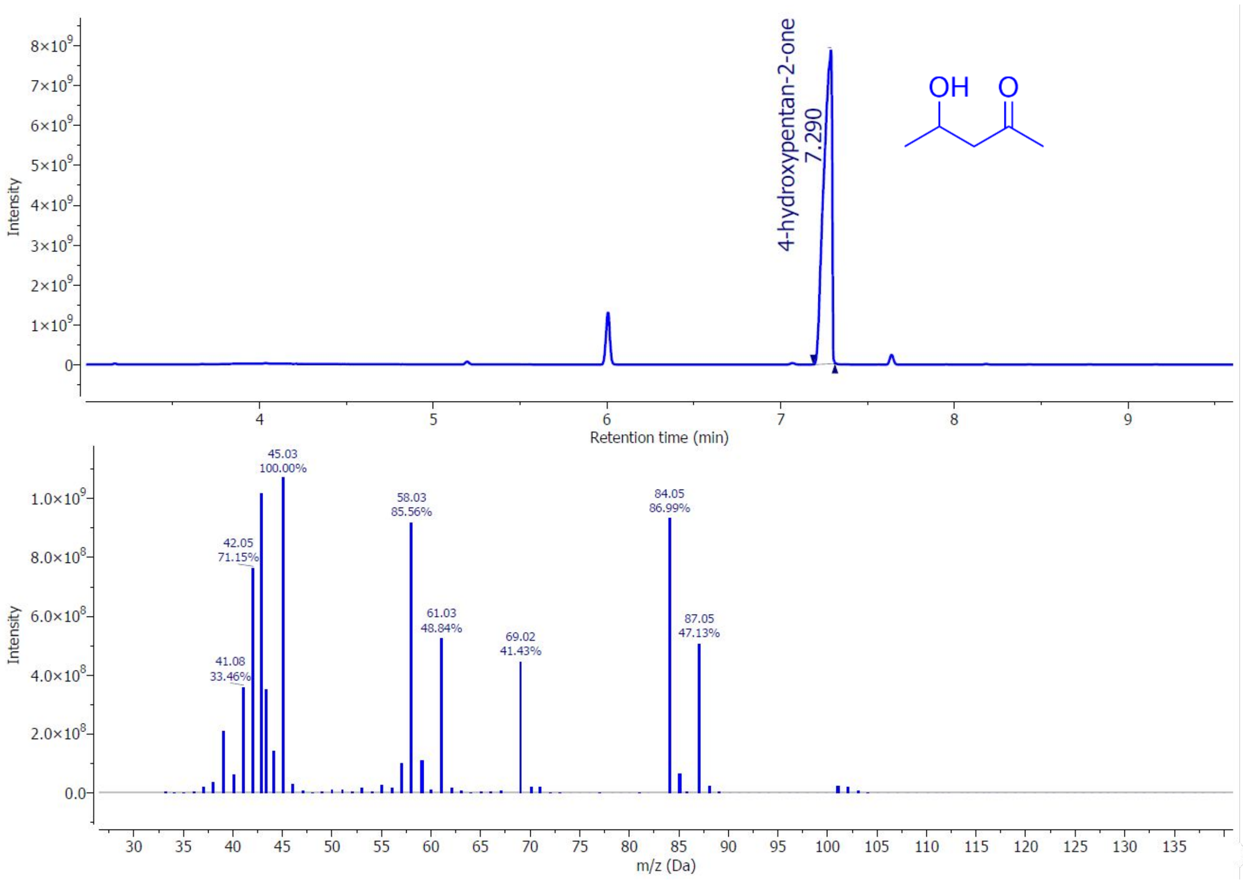

Figure S9. GC-MS analysis of the synthesized 4-hydroxypentan-2-one using DMDO.

The data show the product which was synthesized from the reaction of 2,4-pentanediol with DMDO. This porduct was used in the DSC study (Figure 12).

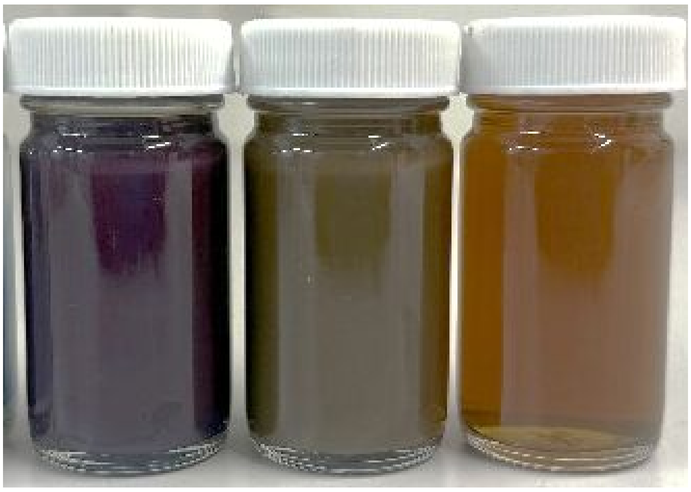

Figure S10. Silver nanoparticles synthesized in 1-decanol using Ag_2O as the precursor. By controlling the reaction time (10-160 min) at 100 °C, the solution undergoes distinct color changes. These physical changes correspond to silver particle with different size, shape, and distance between particles.[2]

Demostration of the influence of the reaction time on the silver particle resulting from reduction of Ag_2O in 1-decanol. By changing the reaction time, the nucleation and growth rates change**.** Higher temperatures generally lead to faster growth (dark colors).[2]

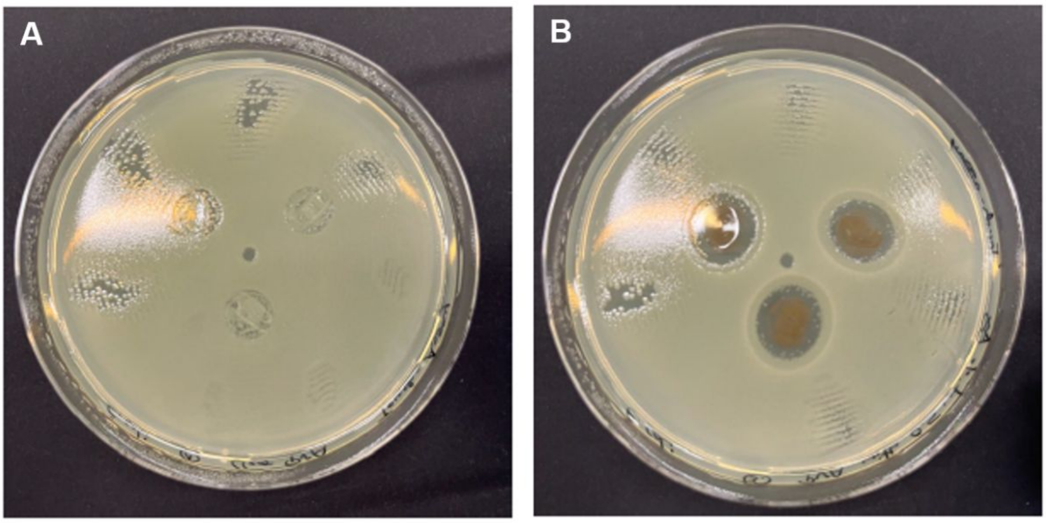

Figure S11. Antimicrobial agar diffusion test against E. coli ATCC 8739 using a camera. (A) A neat PVA control film did not exhibit an inhibition zone, (B) PVA/Ag nanocomposite film (0.5% w/w Ag_2O) demonstrated a clear zone of inhibition.

A digital image of the antimicrobial agar diffusion test.

References:

(1) Koga, N.; Yamada, S.; Kimura, T. Thermal Decomposition of Silver Carbonate: Phenomenology and Physicogeometrical Kinetics. *J. Phys. Chem. C* **2013**, *117* (1), 326–336. https://doi.org/10.1021/jp309655s.

(2) Chhatre, A.; Solasa, P.; Sakle, S.; Thaokar, R.; Mehra, A. Color and Surface Plasmon Effects in Nanoparticle Systems: Case of Silver Nanoparticles Prepared by Microemulsion Route. *Colloids and Surfaces A: Physicochemical and Engineering Aspects* **2012**, *404*, 83–92. https://doi.org/10.1016/j.colsurfa.2012.04.016.
